# Surface plasmon mediated harmonically resonant effects on third harmonic generation from Au and CuS nanoparticle films

**DOI:** 10.1515/nanoph-2022-0630

**Published:** 2023-01-19

**Authors:** Nathan J. Spear, Yueming Yan, Joshua M. Queen, Mahi R. Singh, Janet E. Macdonald, Richard F. Haglund

**Affiliations:** Interdisciplinary Materials Science, Vanderbilt University, Nashville, TN 37235, USA; Department of Physics and Astronomy, Vanderbilt Institute of Nanoscale Science and Engineering, Vanderbilt University, Nashville, TN 37235, USA; Department of Chemistry, Vanderbilt Institute of Nanoscale Science and Engineering, Vanderbilt University, Nashville, TN 37235, USA

**Keywords:** Au nanoparticle, CuS nanoparticle, harmonic generation, harmonic resonance, localized surface plasmon resonance, plasmonic enhancement

## Abstract

A growing class of nonlinear materials employ the localized surface plasmonic resonance (LSPR) of nanoparticles to enhance harmonic generation. Material systems containing harmonically coupled metallic and semiconductor plasmonic nanoparticles have been shown to further increase performance. Here, we explore the effect of dual plasmonic interactions in bilayer CuS and Au nanoparticle films on third harmonic generation (THG). Detuning the CuS LSPR away from the excitation frequency changes the dominant upconversion pathway from THG to multiple photon photoluminescence (MPPL). Changing the size of the Au nanoparticle red shifts the LSPR from the second harmonic of the pump frequency and also eliminates the enhancement effect. When both LSPRs satisfy the harmonic condition, simultaneous excitation of CuS-Au nanoparticle films at the resonant frequency of each nanoparticle species enhances the generation of third harmonic light by sum-frequency generation, suggesting that the enhancement of THG in dually plasmonic nanoparticle films is the result of a cascaded nonlinear mechanism. An analytic model of the interaction between the plasmonic nanoparticles due to incoherent dipolar interactions is also presented. Understanding these processes opens a pathway for developing ultrafast, high-efficiency upconversion thin-film devices by clarifying the conditions that efficiently produce third harmonic generation without background MPPL or additional harmonics.

## 1 Introduction

Metal nanostructures have been widely employed to enhance nonlinear optical processes, such as second harmonic generation (SHG) [1–3], third harmonic generation (THG) [4–6], and multiphoton photoluminescence (MPPL) [7–9]. Metal nanoparticles, with their high electron polarizabilities, intense optical resonances and high surface-to-volume ratios, become effective substrates for optical phenomena that occur on surfaces or in thin films. For example, when excited by an intense light field, the localized surface plasmonic resonance (LSPR) generates gigantic local electric fields, enhancing Raman signals of nearby molecules, or yielding high upconversion efficiency. Plasmonic properties can be varied by material and structural factors [10, 11], or by the creation of bimetallic alloys [12, 13].

Increasingly complex coupled systems have been prepared that demonstrate strong nonlinear response enhancement, especially for coupled semiconductor and metallic nanoparticle systems [14–16]. In such systems, for example, the metal nanoparticles modify the nonlinear response of the semiconductor nanoparticle via coupling between the plasmonic resonance and excitons in the semiconductor [17]. Our complementary approach has been to examine the enhanced harmonic generation observed in a heterostructured film comprising plasmonic semiconductor and plasmonic metallic nanoparticles separated by a thin layer of insulating ligands. In contrast to plasmon-exciton approaches, dually resonant plasmonic systems feature additional local electric field enhancement from both nanoparticles, further increasing harmonic generation efficiency [18, 19].

An emerging class of these biplasmonic structures feature plasmonic resonances that harmonically couple to each other, such that one resonance is energetically located at an excitation fundamental and the other resonance located at a harmonic of the excitation energy [20–22]. These systems have promise as platforms for efficient and thin-film harmonic generators. A CuS nanostructured thin film with uniform nanoparticle-size distribution, prepared by a simple and cost-effective chemical bath deposition method (CBDM), shows high-quality optical and electric properties [23]. The LSPR peak of CuS can be tuned by electrochemical reduction [24]. Many deposition methods have been reported in addition [25, 26].

Copper sulfide belongs to a class of heavily doped colloidal nanocrystals of metal chalcogenides that exhibit both semiconducting and plasmonic behavior. Their plasmonic behavior originates from the collective oscillation of excess free carriers associated with constitutional vacancies, leading to a NIR plasmon resonance. Moreover, the LSPR features of metal chalcogenide nanoparticles are controlled by their geometric parameters, such as size and shape, in addition to the constitutional free carrier density determined by the particular stoichiometric composition and crystal structure [27–29]. Covellite CuS possesses semi-metallic character due to a large concentration of delocalized holes in the valence band from stoichiometric constitutional S^2−^ vacancies. The extinction band of CuS results from the convolution of weak out-of-plane and dominant in-plane dipolar LSPR modes [30, 31]. These modes can be separated by a change in crystal orientation or the incident light polarization [32].

The LSPR of metallic gold arises from the collective oscillation of Fermi-edge electrons. Their resonant energy is tunable from the UV through to the near-infrared predominantly by changing geometry: shells, aspect ratio, shape, and alloying with other elements. Particle size can also tune the LSPR, as red-shifted quadrupolar resonances become increasingly important for large particles [33–35]. The size of the nanoparticle also affects the dephasing time of the collective resonance, with spherical particles larger than 50 nm producing long dephasing times, on the order of tens of fs. Very small nanoparticles, with diameter less than 3 nm, are not large enough to feature near field effects. Instead, excitation decays into hot electrons within 1 fs. Intermediate sizes, such as the 15 nm particles predominantly examined here, feature dipolar LSPR with medium dephasing times that decay into hot electrons [36].

Resonant excitation of the gold LSPR can enhance SHG [37], as demonstrated in various nanostructural designs: split-ring-resonators [38], arrays of gold nanorods [39, 40], a single dimer of gold nanospheres [41], and hybrid plasmon-fiber cavity systems [42]. However, few studies have reported on the enhancement of harmonic generation from Au nanoparticles coupled to harmonically resonant plasmonic semiconductor nanoparticles such as the hybrid Au/CuS nanoparticle films that we explore herein. This combination has the potential for dual plasmonic structures in which the resonances are spectrally located such that a harmonic relationship exists between the excitation energies of their respective LSPRs.

Previously, we observed that Au/CuS bilayer nanoparticle films enhanced the yield of second-harmonic light by a factor of 3.3 compared with the sum of constituent nanoparticles on their own [43]. Additionally, these films exhibited signals from several upconversion pathways, including SHG, THG and MPPL peaks. It was expected that, as a third-order process, THG would be far less intense the second-order SHG; yet, in these initial studies, the third- and second-harmonic signals exhibited similar peak intensities. This observation suggested that plasmonic interactions between Au and CuS nanoparticles were enhancing THG as much or more than the enhancement of SHG, consistent with other plasmonic nanoscale systems with large third-order polarizability and centrosymmetry [44]. Additionally, a THG process derived from plasmonic interactions has the advantage of occurring on an ultrafast timescale, which means that there is no delay due to the thermalization and recombination steps as occurs plasmon-exciton coupled systems. The peak excitation frequency of Au/CuS bilayer heterostructures is also centered near the telecom O- and E-bands, meaning that it can be integrated into thin-film optical modulators for fiber telecommunications making for potentially promising applications of such structures in ultrafast communication technologies.

Here we demonstrate that the dominance of THG or MPPL upconversion mechanisms can be switched by detuning of the CuS or Au plasmon resonance from the harmonic condition. To separate THG from possible degenerate sum frequency generation (SFG), a dual beam setup with both fundamental (frequency *ω*) and second harmonic (frequency 2*ω*) beams was constructed [45, 46]. By changing the ratio of *ω*:2*ω* intensities, we observed for the first-time simultaneous excitation of the LSPR of both nanoparticles in the Au/CuS nanoparticle films. This simultaneous excitation increased the yield of the 3*ω* signal, lowering the detection onset threshold (the lowest power at which signal could be measured above the background). Finally, a dipole-dipole interaction analytic model for the enhancement of THG from Au/CuS bilayer heterostructured films is presented. The behavior predicted by the model closely matches experimentally measured data, suggesting that interactions of coupled CuS and Au surface dipoles is an effective way to understand the upconversion properties of Au/CuS films.

## 2 Results and discussion

Gold nanoparticles were synthesized following literature protocols in several sizes: 3 nm [47], 15 nm [48], and 100 nm [49]. The gold nanoparticles of 3 and 100 nm diameters, which were synthesized in water, were transferred to an organic solvent for film deposition following a transfer ligand-based approach to cap the surfaces with octadecylamine [50]. Likewise, CuS nanoparticles were synthesized using well established approaches leaving them capped with oleylamine. Nanoparticles were deposited sequentially onto glass slides using spin coating to form bilayer films. scanning electron microscopy (SEM) images demonstrated that the deposited disk-shaped CuS nanoparticles had an average diameter of 15 nm—after size selection during the spin-coating deposition process, which selects for the smaller particles [51]. However, there was also a population of disks that were much larger, with diameters on the order of one hundred nanometers.

The structure of the films and their optical properties were confirmed by SEM in secondary electron and backscatter detection modes (Figure S1) and UV–vis–NIR spectrophotometry, respectively. The thickness of the combined films was measured by profilometry to be about 120 nm (Supplementary Figure S5). These diagnostics confirmed the bilayer structure of the nanoparticles on the glass, as well as the presence of the thick, encapsulating layer of oleylamine ligand surrounding the nanoparticles. Due to imperfect packing of the lower (Au nanoparticle) layer, we expect that some CuS nanoparticles, which form the upper layer, will intercalate into the lower layer. We do not expect this to have a significant impact on the interaction between the two plasmonic nanoparticles, as previous experiments have demonstrated that switching the ordering of Au and CuS (top vs. bottom) of the two layers had no effect on upconversion yield. Moreover, the gap size between nanoparticles in the packing of the lower layer is much smaller than the beam waist of the focused beam, meaning that the effect of the imperfectly packed zones is averaged out over the sampling area. The ligand layer is critical to preventing the nanoparticle layers from coming in direct contact; were this to occur, then the nanoparticles would quench each other’s LSPRs [52], preventing harmonic coupling. In the bilayer films described here, the oleylamine layer separates each nanoparticle from its neighbors by a few (3–5) nanometers [53], forming a somewhat diffuse layer of nanoparticles whose three-dimensional spatial density depends on the number of oleylamine molecules that intercalate between the nanoparticles. Since particles are approximately 15 nm, we can conclude the films are of order of five nanoparticles of Au and CuS thick. While there is considerable variation in the inter-nanoparticle spacing, over the lateral extent of the laser focal spot (10 μm) the average inter-particle spacing homogenizes the upconversion response at various sampling points across the film.

**Figure 1:**
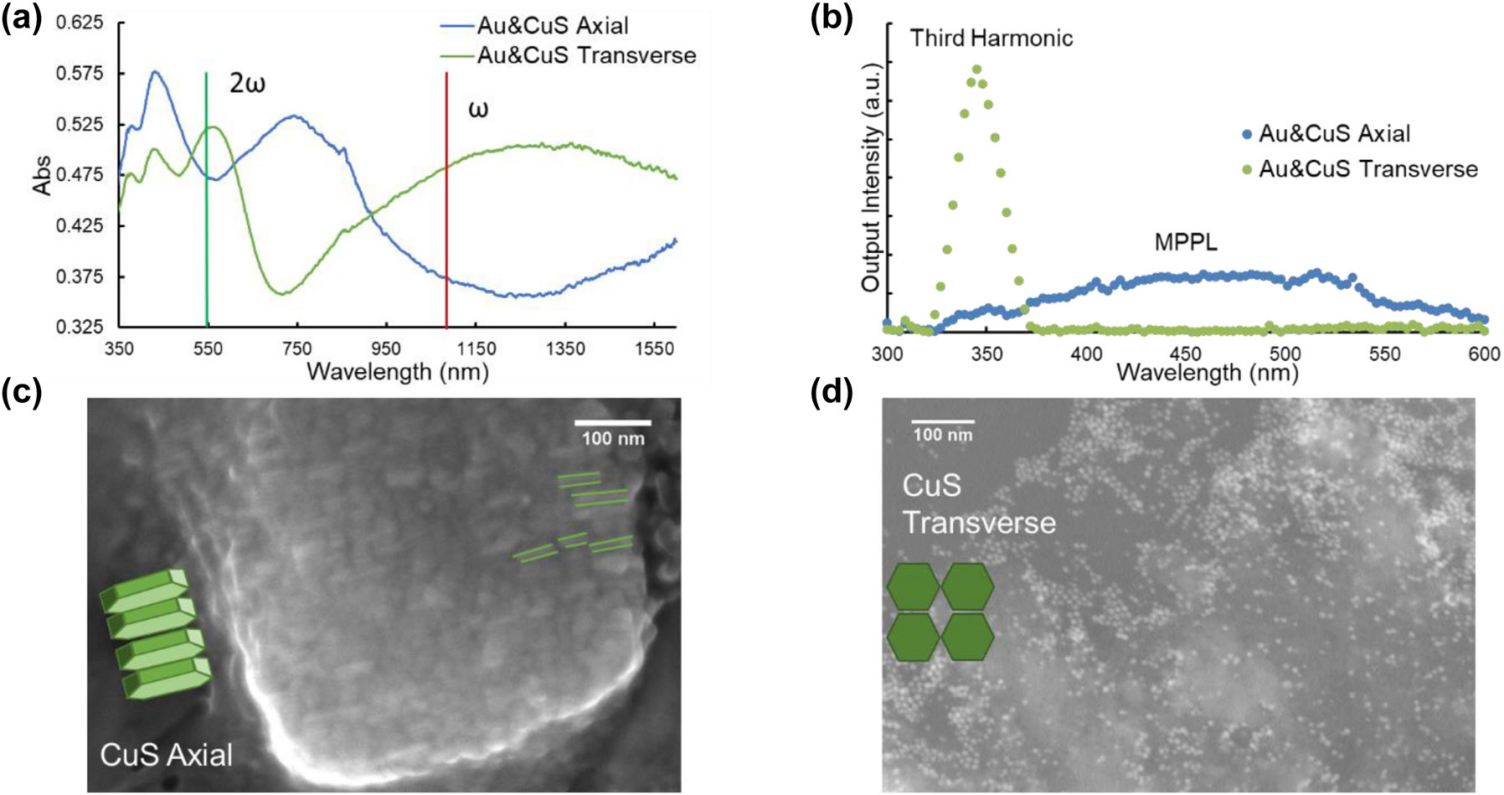
Effects of CuS nanodisk orientation on film absorption and upconversion mechanism. (a) UV–vis–NIR spectrophotometry of two heterostructure films deposited on glass microscope slides: Au with CuS in axial orientation (blue), Au with CuS in transverse orientation (green). (b) Spectra of output light produced by the two nanoparticle heterostructures. (c) SEM images of CuS nanoparticles with axial orientation produced by drop casting. The green lines highlight the edges of select nanodisks as a guide to the eye. (d) SEM images of CuS nanoparticles with transverse orientation produced by spin coating.

The processing conditions under which CuS nanoparticles were deposited had a large impact on upconversion properties. When nanoparticles were drop-cast onto a substrate (with Au nanoparticles already situated thereon) and allowed to dry into a layer, the LSPR was blue shifted compared to the colloidal measurement in solution. The SEM and in-plane XRD measurements indicated that the CuS disks would orient face-to-face and lie in stacks on their edges. The particle stacking caused the blue-shifted, out of plane (axial) mode of the plasmon resonance to become the dominant resonance (Figure 1a) [32]. Unlike spin-coating, drop casting the CuS nanodisks also did not select for any particular size, leading to a more heterogeneous size distribution of nanoparticles in the film, as evident from the lengths of the particle edges visible in Figure 1c. This blue shift caused the LSPR of CuS to no longer align with the excitation frequency of the laser. Thus, this change in processing effectively detuned the surface plasmon from the resonant condition. Upon stimulation by a 1050 nm laser, the detuned sample exhibited the broad emission peak characteristic of MPPL (Figure 1b). Without the resonant enhancement from the surface plasmon at the fundamental frequency, harmonic generation was not efficient enough to be detected at the excitation intensities available in our experiment (6.7 GW/cm^2^).

In contrast, when CuS nanoparticles were added to the films by spin-coating, SEM (Figure 1c and d) indicated that the particles were mostly aligned with basal planes parallel to the surface. Additionally, the population of large-diameter nanoparticles that was present in solution was not evident on the substrate after spin coating, thus narrowing the size distribution. The absorption peak centered at 1300 nm indicated that the in-plane (transverse) mode dominated the profile of the LSPR. With laser excitation at 1050 nm, a signal centered on 3*ω* was measured, although it was not possible with this experiment alone to determine of this signal resulted from THG directly, or a combination of SHG and SFG (cascaded THG). While THG typically has a narrow line width dictated by the laser characteristics, in these experiments the entrance slit of the monochromator was set to its maximum value to maximize the signal-to-noise ratio, resulting in an artificially broadened peak. In short, the tuning and detuning of the CuS plasmon resonance indicates that coincidence with the laser fundamental is necessary to observe the strong 3*ω* signal indicative of harmonic generation in bi-plasmonic systems, rather than the broad output of MPPL.

**Figure 2:**
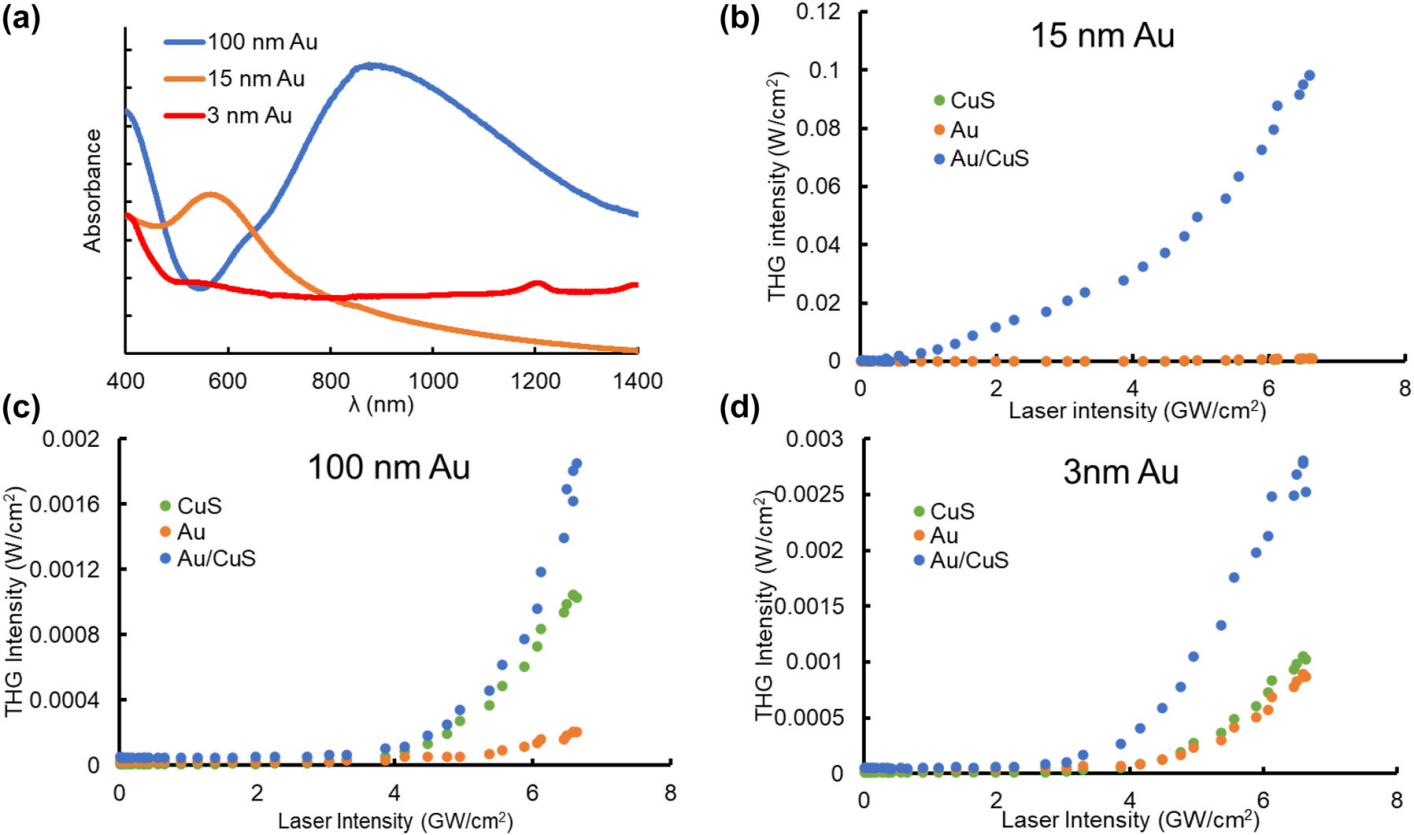
Effects of Au nanoparticle size on THG enhancement due to detuning of harmonic resonance. (a) UV–vis–NIR spectrophotometry of three size Au films deposited on glass microscope slides. Intensity of the THG signal generated by 1050 nm excitation as a function of the input laser intensity for films with different Au nanoparticle diameters and CuS in the transverse orientation, (b) *d*_Au_ = 15 nm, (c) *d*_Au_ = 100 nm, (d) *d*_Au_ = 3 nm.

Tuning the plasmon resonance of Au nanoparticles was also integral to the efficiency of harmonic generation. Spin coated films were prepared with Au nanoparticles of 3, 15, and 100 nm diameters. The intensity of third harmonic light produced by these films was measured, then identical layers of CuS were coated atop the Au nanoparticle layers to compare the extent to which the LSPR of the Au nanoparticles of differing sizes interact with the LSPR of CuS. The selected sizes of Au nanoparticles were chosen because of their have significant differences in their collective plasmon oscillations (Figure 2a). The 15 nm Au nanoparticles exhibited narrow, dipolar LSPR with its peak centered at *λ* = 550 nm. This resonance corresponds most closely to the second harmonic of the pump laser (and thus the second harmonic of the plasmon resonance of the CuS nanoparticles). The 100 nm Au nanoparticles had a broader resonance than the 15 nm particles, and the peak absorption wavelength was shifted towards the red in addition to longer dephasing times (tens of fs) and broadband scattering. Conversely, 3 nm diameter Au nanoparticles have a very low absorbance cross section and such short dephasing times (1 fs) that the excitation quickly decays into hot electrons, precluding the formation of an LSPR absorbance peak [54, 55].

The efficiency for generating 3*ω* light (350 nm) from heterostructured films containing 3, 15, and 100 nm diameter Au nanoparticles varied widely. Films containing 15 nm Au nanoparticles and CuS nanoparticles demonstrated the strongest signal at the third harmonic, with a 20-fold enhancement over the sum of the 3*ω* light produced by Au and CuS separately (Figure 2b). The LSPR of the 15 nm Au nanoparticles closely aligns with the harmonic condition (Au LSPR at 2*ω*) that produces enhanced harmonic generation.

In stark contrast, films with 100 nm diameter Au particles produced a much more modest amount of third harmonic light. Indeed, there appeared to be no enhancement effect, as films with 100 nm Au and CuS nanoparticles produced only as much 3*ω* signal as the sum of their unenhanced yields. The LSPR of the 100 nm Au nanoparticles is detuned substantially from the harmonic condition, and this appears to inhibit harmonic coupling of the CuS and Au plasmonic resonances (Figure 2c) which is needed for efficient production of 3*ω* light. Similarly, films containing 3 nm Au and CuS nanoparticles also only generated as much 3*ω* light as the sum of each nanoparticle layer separately, once again suggesting that there is no plasmonic enhancement by bringing nanoparticles into proximity unless they also satisfy the harmonic condition. The 3 nm particles feature very low LSPR absorbance, and so again, the harmonic condition between the CuS (LSPR at laser fundamental *ω*) and Au (at 2*ω*) seems to be necessary for enhanced the signal at 3*ω*. Notably, third-order dependence was measured for samples at all sizes of Au nanoparticles, except the smallest nanoparticles (Supplementary Figure S7). On their own, the 100 nm Au nanoparticles generated less 3*ω* light than 15 nm Au particles by a factor of five at the highest excitation intensity. 3 nm diameter Au nanoparticles also produced a lesser quantity of upconverted light than 15 nm diameter particles by a factor of 1.5. Taken together, these results strongly suggest that enhancement of 3*ω* signal only occurs when the plasmon resonance of the Au is energetically coincident with the second harmonic of the pump laser. The enhancement of harmonic generation, then, is a resonant effect mediated by the LSPR and thus is size dependent, rather than having its origins in crystallographic or macroscale material properties.

Having confirmed the essential role of the harmonic positioning of the LSPR in Au and CuS nanoparticles, the question remained how the upconversion to the third harmonic was occurring in the bilayer films, as two separate mechanisms appeared likely. Ambiguity existed between a direct THG process (*ω* + *ω* + *ω* = 3*ω*) and a degenerate SFG process (*ω* + *ω* = 2*ω* then *ω* + 2*ω* = 3*ω*); a cascaded THG process would explain why the third harmonic signal was similar in intensity compared to the second harmonic in our previous study due to the second-order nature of the SFG process [43]. Both cascaded and direct THG yields follow a third-order nonlinearity; thus, further experimentation was required to disambiguate the two processes by testing the possibility of SFG in the nanoparticle bilayer films directly using separate *ω* and 2*ω* beams [56].

**Figure 3:**
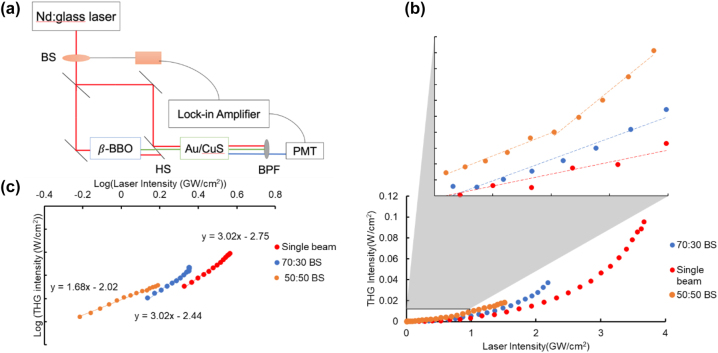
Sum frequency generation from Au/CuS bilayer films by dual-beam pumping. (a) A simplified diagram of the experimental dual-beam setup. BS, beam splitter; HS, harmonic separator, BPF = 350 nm bandpass filter. Red line is 1050 nm fundamental beam, green is 525 nm (2*ω*) beam, and blue 350 nm (3*ω*) beam. (b) Intensity of THG output generated by hybrid films as a function of fundamental beam intensity after beam splitter for different *R*:*T* ratio beam splitter: 100:0 (red—power of 2*ω* 0 μW), 70:30 (blue—power of 2*ω* beam 185 μW), 50:50 (orange—power of 2*ω* 300 μW). (c) Double-logarithmic plots of THG intensity as a function of pump laser intensity for the data of (b).

The films were pumped by both 1050 nm (*ω*) and 525 nm (2*ω*) light by using a beam splitter and a high-efficiency frequency doubling crystal, β-barium borate (BBO), in an approach similar to the demonstration of SFG in other nanostructured materials (Figure 3a) [45]. This approach decouples the SHG and SFG steps in the cascaded THG process, allowing direct testing for the probability of exciting the second (SFG) step in cascaded THG. Simultaneous excitation at 1050 nm and 525 nm directly induces surface plasmon resonances in the CuS and Au, respectively. With both plasmon resonances actively excited, the efficiency of 3*ω* light generation increased substantially compared to single-beam pumping at the same intensity (Figure 3b), due to the addition of a 3*ω* signal from SFG in addition to the direct THG output. Notably, no measurable signal was detected from 2*ω*-only excitation, likely due to the low intensity of the 2*ω* beam with losses during the upconversion in BBO.

Simultaneous excitation at *ω* and 2*ω* enhanced the yield of 3*ω* light in these bi-plasmonic systems across a range of *ω* and 2*ω* intensities. These experiments were performed by decreasing the reflectance::transmittance ratio in the beam splitter, which in effect lowers the relative intensity at 1050 nm while increasing the relative intensity at 525 nm. After beam splitting, the intensity of the upconverted beam at 525 nm was held steady, while the intensity at 1050 nm was attenuated with a polarizer.

Adding excitation at 2*ω* caused an increase in the light yield at 3*ω*. That is, for a given input intensity at *ω*, the amount of 3*ω* light produced is greater for dual beam-excitation than for pumping at *ω* only. Indeed, even comparing dual-beam excitation with different mixes of components, the mix with more 2*ω* excitation produced more signal at 3*ω* (50:50 BS curve is above 70:30 and 100:0 at the same laser intensity).

The use of the 50:50 split in the excitation beam stands out, as it had an order of only 1.68 with respect to the *ω* intensity (Figure 3c). This configuration has overall low power as a result of losses in the beam splitter and during the upconversion process of half the intensity to 2*ω*. At such low power, the light produced at 350 nm results from a mixture of SFG (first order with respect to *ω*) and MPPL (high order —previously reported as ∼6 [43]) mechanisms, yielding the overall measured order of 1.68. Under more intense conditions, seen with the other beam splitters, cascaded THG dominates the smaller MPPL contribution. The presence of the MPPL signal is discussed and demonstrated in Supplementary Figure S4. With the feasibility of SFG in CuS-Au nanoparticle bilayer films established, the observed enhancement of THG due to the presence of a harmonic plasmon resonance suggests that a cascaded mechanism of SFG seeded by SHG is responsible, rather than enhancement of a direct THG process.

To test this explanation, we built an analytical model based on a dipole-dipole interaction and observed its correspondence with experimental THG measurements. To set about building an analytic description of the harmonic generating properties of Au-CuS nanoparticle bilayer films, we considered the effect of the electric field of each nanoparticle upon the other nanoparticle. In an approach akin to that taken for similar heterogenous nanoparticle ensembles [16, 57], coherence between the LSPR fields was not considered because the dephasing times of the plasmons are much shorter than the duration of the pump pulse, meaning that the resonances very quickly lose coherence and thermalize—due to electron-electron collisions—once the pump pulse ends.

The full derivation and model details are available in Supporting Section S2. In brief, the far-field intensity of the third harmonic (*I*_THG_) output by the bilayer system will be the sum of the 3*ω* signal originating from each component.

(1) $$I_{\text{THG}}^{\text{hybrid}}=I_{\text{THG}}^{\text{Au}}+I_{\text{THG}}^{\text{CuS}}$$

Each component in turn, will be dependent upon the intensity of the electric fields around them. For example, the CuS nanoparticles will have terms for fields from the laser pump beam (*I*_p_^3^) and the LSPR field of the Au nanoparticles (*I*_Au_^3^), but also the cross terms between the Au LSPR and the pump beam (*I*_p_^2^*I*_Au_ and *I*_p_*I*_Au_^2^). Scaling factors Π_Au/CuS_ are needed to account for the extinction coefficients of the particles at the pump frequency (we expect Π_Au_ to be much smaller than Π_CuS_ at *ω* and vice versa at 2*ω* judging from their absorbance spectra). Thus, the general statement for THG from the Au-CuS nanoparticle system is:

(2) $$\begin{array}{ll} I_{\text{THG}}^{\text{hybrid}} & =\left[\alpha_{\text{cst}}^{\text{Au}}\Lambda_{\text{au}}^{6}{\left|F_{\text{ppp}}^{\text{Au}}\right|}^{2}\left\{{I_{p}}^{3}+9{\Pi_{\text{CuS}}}^{2}{I_{p}}^{2}I_{\text{CuS}}\right.\right.\\ & \;+\left.9{\Pi_{\text{CuS}}}^{4}{I_{\text{CuS}}}^{2}I_{p}+{\Pi_{\text{CuS}}}^{6}{I_{\text{CuS}}}^{3}\right\}+\alpha_{\text{cst}}^{\text{CuS}}\Lambda_{\text{CuS}}^{6}\\ & \;\times {\left|F_{\text{ppp}}^{\text{CuS}}\right|}^{2}\left\{{I_{p}}^{3}+9{\Pi_{\text{Au}}}^{2}{I_{p}}^{2}I_{\text{Au}}+9{\Pi_{\text{Au}}}^{4}\right.\\ & \;\times \;\left.\left.{I_{\text{Au}}}^{2}I_{p}+{\Pi_{\text{Au}}}^{6}{I_{\text{Au}}}^{3}\right\}\right] \end{array}$$

(3) $$E_{\text{spp}}^{\text{Au}}=\Pi_{\text{Au}}E_{P}\;\;\;\;\;\;\Pi_{\text{au}}=\frac{V_{\text{Au}}g_{l}}{4\pi r^{3}}\zeta_{\text{Au}}$$

(4) $$E_{\text{spp}}^{\text{CuS}}=\Pi_{\text{CuS}}E_{P}\;\;\;\;\;\;\;\Pi_{\text{cus}}=\frac{V_{\text{CuS}}g_{l}}{4\pi r^{3}}\zeta_{\text{CuS}}$$

*α*_cst_, Λ, and *F*_ppp_ are constants relating the charge carrier density and mobility of Au and CuS as well as nanoparticle size and spacing to the nonlinear susceptibility of these materials Equation (5).

(5) $$\chi_{\text{ppp}}^{\text{Au}}=\frac{2\mu_{21}^{3}p_{\text{Au}}}{V_{\text{Au}}\in_{0}\hbar^{3}}\left(\Lambda_{\text{Au}}^{3}F_{\text{ppp}}^{\text{Au}}\right)$$

Equation (2) implies a bilateral relationship between the two nanoparticles, with each nanoparticle enhancing the local electric fields for the other nanoparticle. Thus, we see that Equation (2) is composed of two components, one of which represents THG from the Au nanoparticles—with intensity terms from the pump beam and the local field of the CuS LSPR—and the other of which represents THG from CuS nanoparticles, again with intensity terms from the pump beam and the Au LSPR.

Under single beam excitation at 1050 nm (*ω*), the absorbance of the Au plasmon is negligible, Equation (2) simplifies to:

(6) $$\begin{array}{ll} I_{\text{THG}}^{\text{hybrid}} & =\left[\alpha_{\text{cst}}^{\text{Au}}\Lambda_{\text{au}}^{6}{\left|F_{\text{ppp}}^{\text{Au}}\right|}^{2}\left\{{I_{p}}^{3}+9{\Pi_{\text{CuS}}}^{2}{I_{p}}^{2}I_{\text{CuS}}\right.\right.\\ & \;+\left.9{\Pi_{\text{CuS}}}^{4}{I_{\text{CuS}}}^{2}I_{p}+{\Pi_{\text{CuS}}}^{6}{I_{\text{CuS}}}^{3}\right\}\\ & \;+\left.\alpha_{\text{cst}}^{\text{CuS}}\Lambda_{\text{CuS}}^{6}{\left|F_{\text{ppp}}^{\text{CuS}}\right|}^{2}\left\{{I_{p}}^{3}\right\}\right] \end{array}$$

because the Π_Au_ terms are near zero due to how far off 1050 nm stimulation is from the Au resonance. Under dual-beam excitation all of the terms of Equation (2) are nonzero, and it is the additional terms containing Π_Au_ which account for the increase in measured THG in the dual-beam mode.

The importance of the harmonic condition between the LSPR of Au and CuS nanoparticles is explicable with the simple model of the superposition of two waves (with the same group velocity) and different frequencies [58], *f*_1_ and *f*_2_. This approach models near-field coupling between the respective LSPR of the nanoparticles as opposed to the treatment provided in the SI, which considers the LSPRs as acting incoherently upon each other. This resulting superimposed wave has greater amplitude than the input waves (Equation (7)), in which the amplitude term (*E*_*m*_) has doubled.

(7) $$\begin{array}{ll} E\left(x,t\right) & =E_{m}\cos\left(k_{1}x-\omega_{1}t\right)+E_{m}\cos\left(k_{2}x-\omega_{2}t\right)\\ & =2E_{m}\cos\left[\frac{k_{1}-k_{2}}{2}x-\frac{\omega_{1}-\omega_{2}}{2}t\right]\\ & \;\times \cos\left[\frac{k_{1}+k_{2}}{2}x-\frac{\omega_{1}+\omega_{2}}{2}t\right] \end{array}$$

In the resulting expression, the amplitude envelope of the resulting oscillation is controlled by the first cosine term. This means that the amplitude profile (beat frequency) is largely controlled by the difference in frequency, consistent with the rotating wave approximation in nonlinear optics, invoked above (Equation (8)) [59].

(8) $$f_{\text{beat}}=\left(f_{1}-f_{2}\right)$$

Thus, when the resonant frequencies of the two oscillations are in a harmonic relationship (*f*_*1*_ = *2f*_*2*_) the beat frequency is the same as the frequency of the fundamental oscillation (*f*_beat_ = *f*_*2*_). Due to the increase in amplitude of the superimposed wave, this has the effect of enhancing the electric field intensity. Notably, if the resonances are tuned away from the harmonic condition, then the beat frequency will not match the fundamental frequency and would not enhance the excitation intensity as effectively, as there lacks coherence between the resonant mode and the excitation pulse. If we apply this approach to the plasmonic resonances of Au and CuS nanoparticles in a bilayer film, treating the LSPR as dipolar oscillations (as above) each of which are actively being pumped at their respective resonant frequencies— as is the case for the simultaneous dual beam excitation condition, but not in the 1050 nm only excitation—then the beat frequency is the same as the resonant frequency of the CuS nanoparticle (*f*_beat_ = 285.5 THz, 1050 nm). Thus, the amplitude of the electric field at *ω* is enhanced by the presence of the Au LSPR, which concomitantly increases the quantity of THG from the CuS.

**Figure 4:**
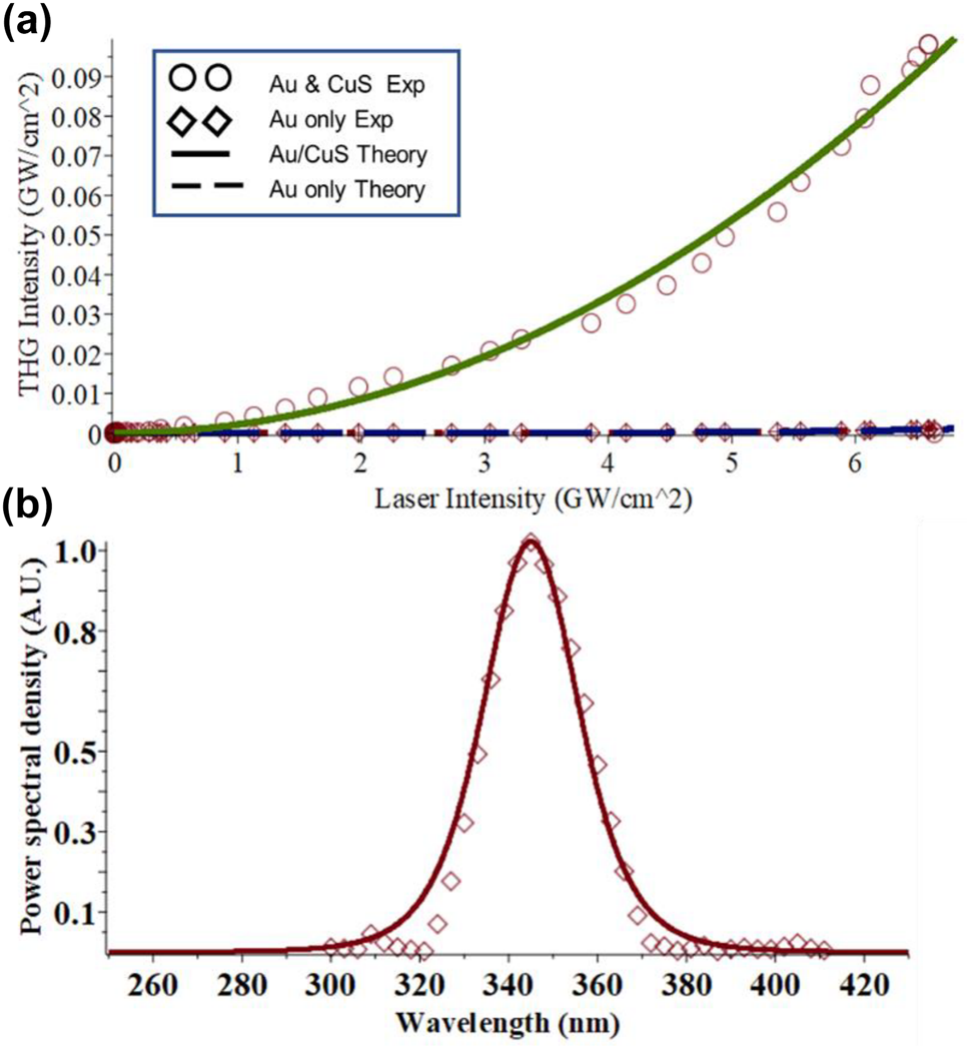
Correlation between experimental results and predicted values from dipole–dipole analytic model. (a) THG signal as a function of input intensity from predicted from analytic model for: 15 nm diameter Au and CuS nanoparticle hybrid film (solid green) and Au nanoparticle only (dashed purple). Experimental data (Figure 2b) from bilayer films are plotted as circles and data from single species films with diamonds. (b) The predicted output intensity of upconverted light at various wavelengths, diamonds are experimental data from Figure 1b. Line theoretical fit centered on 3*ω* with a fitting full width at half max of 25 nm.

The upconverting properties of the Au/CuS bilayer heterostructure are accurately predicted by the model. In particular, the third harmonic enhancement effect in samples containing 15 nm Au nanoparticles (as measured experimentally) were reproduced by plotting Equation (2) with Π_Au_ and Π_CuS_ as fitting parameters (Figure 4a). The validity of the obtained values these coupling constants was confirmed by separate calculation from the known values of the physical constants (Figure S5). We see that the simple addition of signal from films containing Au and CuS separate from each other, without the *I*_au_ and *I*_CuS_ terms that come from the local field of one nanoparticle’s plasmonic resonance acting on another nanoparticle, is lower than the combined film, in which the nanoparticles interact. Indeed, the relative quantity of enhancement closely matches experimentally measured increase from having both nanoparticles. The spectral width of the output THG signal agrees with the measured values (Figure 4b) once a width factor of 25 nm was added to account for broadening due to the monochromator slit width. Using the spectral profile of the pump laser and plugging the arbitrary intensity thereof at a variety of wavelengths into Equation (1), then plotting the THG output intensity for these wavelengths, the spectral width of the experimental results was closely matched.

## 3 Conclusions

Thin bilayer films containing Au and CuS nanoparticles, with surface plasmon resonances at the frequency of a pump laser (CuS) and at the second harmonic (Au), respectively, exhibited enhanced THG over the individual films as a result of dipole-dipole, incoherent interactions between the nanoparticles. Using the dipole approximation, an analytic model that accounts for both the action of the Au LSPR on harmonic generation in CuS and the action of the CuS LSPR on harmonic generation in Au accurately predicts the upconversion properties of these bilayer nanoparticle films. Additionally, the process–property relationships between the deposition-controlled orientation of the CuS nanoparticles and the output spectrum were explored. It was found that CuS nanoparticles, stacked face-to-face (with their edge facing out), showed a blue-shifted LSPR compared to face-up nanoparticles. This difference in excitation energy corresponded to the disappearance of the 3*ω* signal in the output spectrum, indicating that the LSPR of the nanoparticles was critical to the upconversion mechanism. Similarly, shifting the Au plasmon resonance by changing the nanoparticle diameter in different bilayer films shows that the enhancement effect due to the Au nanoparticle can be eliminated if the resonance of the particles is not resonant with second harmonic of the pump beam. This also supports the assertion that the interactions between the Au and CuS nanoparticles in these films are mediated by their LSPRs. Moreover, direct excitation of the Au LSPR at 2*ω* by simultaneous excitation at the CuS plasmon resonance at *ω* demonstrated efficient sum frequency generation This suggests that the plasmon resonance of Au enhances the generation of 3*ω* light in a cascaded SHG-SFG process.

To further explore the fundamental mechanisms that govern interactions between harmonically resonant plasmonic nanoparticles, the effect of the properties of the gap between the two nanoparticle layers should be investigated. Directly probing the nanoparticle interface with localized measurement techniques such as near-field spectro-microscopy will potentially yield insights into the coupling mechanism that governs the enhanced harmonic generation. Off-resonant excitation from an optical parametric amplifier (OPA) would allow us to explore the various upconversion mechanisms that these films, and possibly demonstrate switching between generation of harmonics and MPPL. Finally, the concept of harmonic interactions between plasmonic nanoparticles for upconversion can be extended to three-plasmon systems or engineered multilayers to increase higher-order harmonic generation and overall efficiency of these systems.

## 4 Experimental section/methods

### 4.1 Nanoparticle synthesis and film deposition

Nanoparticles of CuS and Au were synthesized as described in previous reports [43, 47, 49]. For the 3 and 100 nm diameter Au nanoparticles, which were synthesized in H_2_O, an additional ligand exchange step was necessary to prepare them for film deposition. In this procedure, 400 mg of octadecylamine (Sigma-Aldrich, 90%) was dissolved 10 mL of CHCl_3_ with 1 mL of Dodecanethiol (Sigma-Aldrich, 98%) and placed in a separation funnel. To this solution, 20 mL of as synthesized aqueous colloidal Au was added and vigorously mixed. The layers separated after 3 min and the nonpolar layer (now containing the Au nanoparticles) was extracted. This colloidal suspension was then cleaned by a centrifugation procedure in which 35 mL of ethanol were added and then centrifuged at 8000 rpm for 5 min. The supernatant was decanted, and the pellet of nanoparticles was redissolved in a small amount of toluene. This procedure was performed twice. Deposition of nanoparticles was performed with a Ni-Lo scientific Ni-Lo 5 spin coater. To prepare the nanoparticle suspension for deposition, excess solvent was removed with a rotary evaporator until the liquid was reduced to a dark ink. The concentrated nanoparticle suspension was then pipetted onto a glass slide until the substrate was completely covered, (1 mL for a 2 × 2 cm section) and spun at 600 RPM for 30 s or until dry. The nanoparticle loading on the slide was controlled during the concentration step, with lesser nanoparticle concentrations producing fewer nanoparticles in film. For the deposition of CuS, it was found that the addition of a small amount of oleylamine (3%vol) prior to concentration and deposition improved the quality of the final film and prevented cleaning of the already deposited Au nanoparticles off the substrate. Structure of the nanoparticle films was confirmed with scanning electron microscopy (SEM) (Zeiss Merlin SEM) at 1.10 kV with the InLens secondary electron detector and at 5 kV with the NTS-BSD backscatter detector. Powder X-ray diffraction (XRD) patterns were acquired both in and out of the plane of the film in a Rigaku SmartLab X-ray diffractometer with a CuKα source and a D/teX Ultra 250 detector (out of plane) and an SC-70 detector (in plane). The operating voltage was 40 kV and current were 44 mA. Samples were prepared by drop casting concentrated nanoparticle colloids onto glass microscope slides.

### 4.2 Optical measurements

Extinction spectra of the nanoparticle film samples were acquired in a Jasco V-670 UV–vis–NIR spectrophotometer with an integrating sphere from 350 to 1800 nm. Optical measurements were carried out using the nonlinear microscope depicted in Figure 3a. A femtosecond mode-locked Nd:glass laser (Time-Bandwidth GLX-200 oscillator) provided pulses of center wavelength 1050 nm at an average power of 230 mW and 177 fs duration with a repetition rate of 100 MHz and an energy per pulse of 3 nJ. The laser beam was mechanically chopped at a frequency of 265 Hz, with a duty cycle of 20%, thus, each exposure of the sample lasts roughly 750 μs. Repeated exposures produce an annealing effect on the film [46], but a steady state was reached after approximately 5 min of exposure, permitting stable measurement condition. The pump beam was split into a path that proceeds directly to the sample, and one that is upconverted to 2*ω* by beam splitters with various *R*:*T* ratios (Thorlabs BST11 70:30 (*R*:*T*) and BSW11 50:50 (*R*:*T*) UV Fused silica plate beam splitters). The transmission beam was upconverted with a 0.5 mm thickness β-Barium Borate (BBO) crystal, and then the unconverted fundamental frequency was removed by a harmonic separator (Optosigma YHS-25.4C05-1064). The two beams were then focused onto the sample to form a beam waist of 10 μm, ensuring by the construction that the path lengths through which they traveled were identical. To determine the order of the dependence of THG on fundamental pump intensity, we placed a rotational polarizer on the *ω* beam. Using this, the laser power was adjusted by varying the angle between the output polarization of the pump laser and the rotational polarizer. The pump power was measured in a Thorlabs S130C power meter with a PM100D readout. The THG signal was isolated from the other upconverted light signals using a band pass filter (Optosigma VPF-25C-10-25-350). Our detector was a solid-state photomultiplier tube (PMT, Hamamatsu, R9875U, NMA0340) operating at 1.1 kV for ultraviolet light detection. A Newport 1/4 m 74100 Monochromator and the PMT detector were used to collect the output spectrum of the nanoparticle heterostructures.

## Supplementary Material

Supplementary Material Details

## Supplementary Material

The article contains supplementary material (https://doi.org/10.1515/nanoph-2022-0630).
