# Supplementary material for: Surface plasmon mediated harmonically resonant effects on third harmonic generation from Au and CuS nanoparticle films
Source: Nanophotonics. 2023 Jan 19;12(2):273–84. doi: 10.1515/nanoph-2022-0630 (PMC11501534; doi:10.1515/nanoph-2022-0630)
Supplement: Supplementary file 1 — Supplementary Material Details [file j_nanoph-2022-0630_suppl.docx]

Supporting Information

Surface Plasmon Mediated Harmonically Resonant effects on Third Harmonic Generation from Au & CuS Nanoparticle Films

Nathan J. Spear & Yueming Yan, Professor Mahi Singh, Joshua M. Queen, Professor Janet E. Macdonald, and Professor Richard F. Haglund*

**
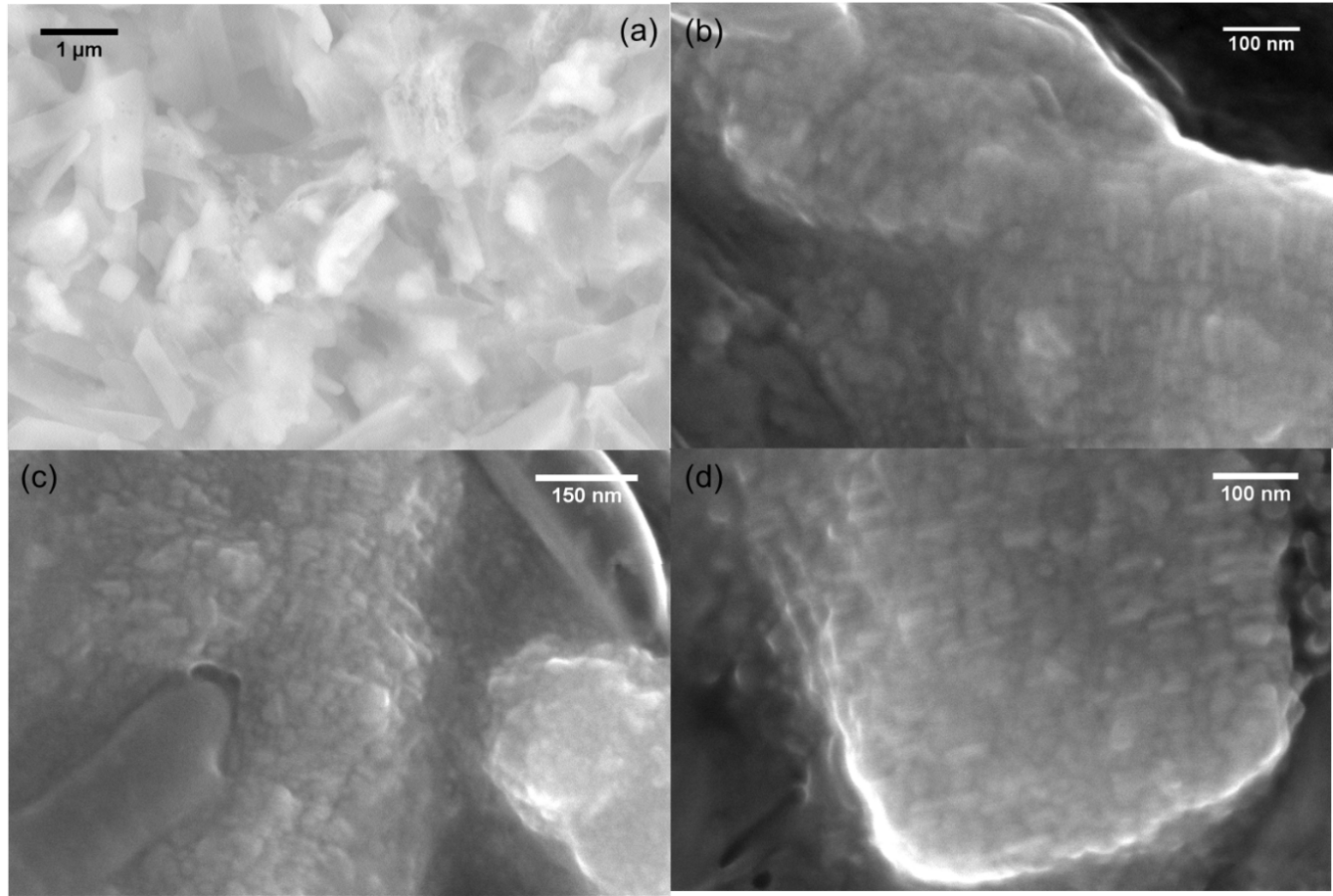
**

**
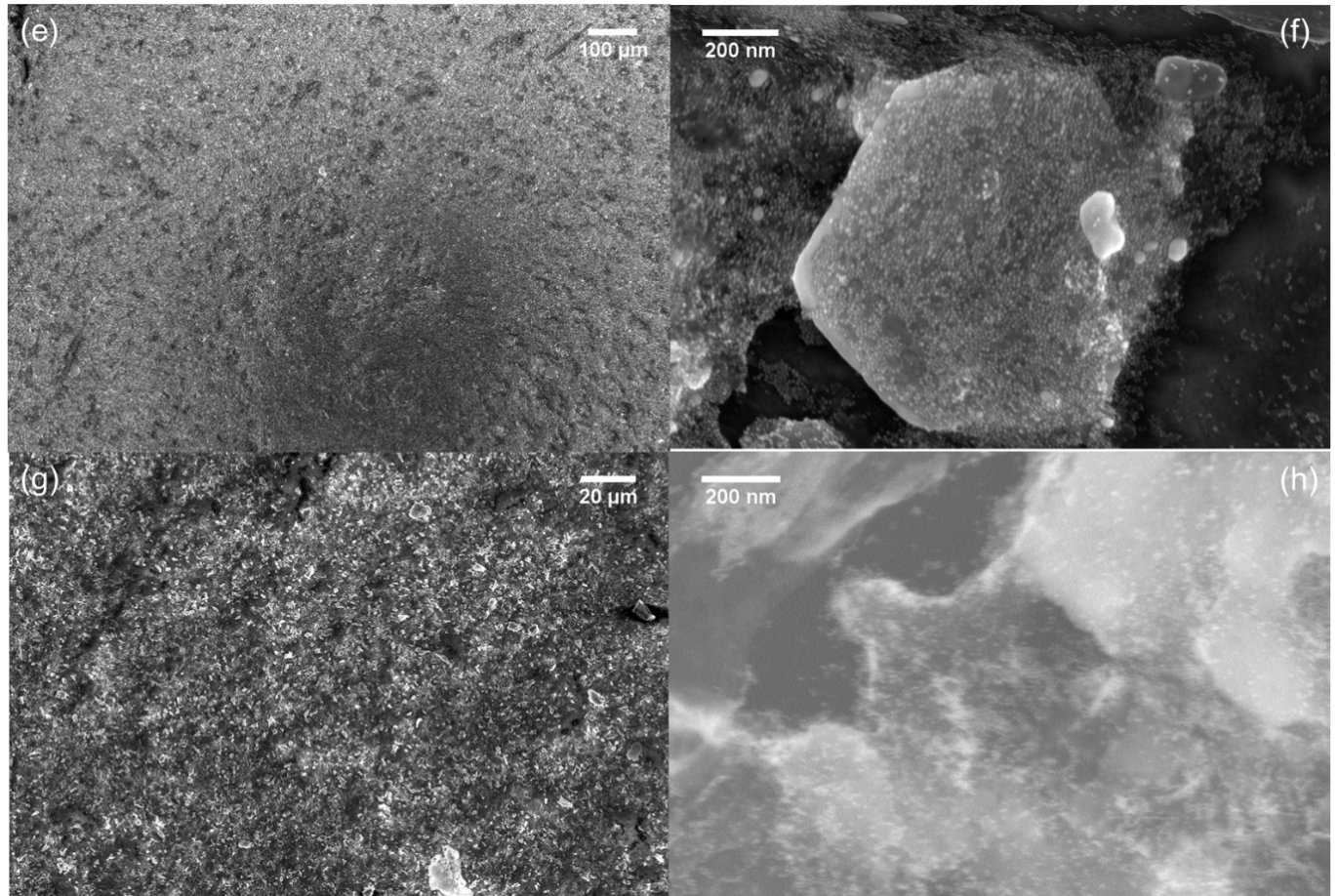
**

**Figure S1.** Scanning electron micrographs of Au-CuS nanoparticle films. (a-d) Samples with CuS in the axial orientation collected with a mixture of backscattered and SE2 electrons. (e-h) CuS in the transverse orientation collected with SE2 electrons.


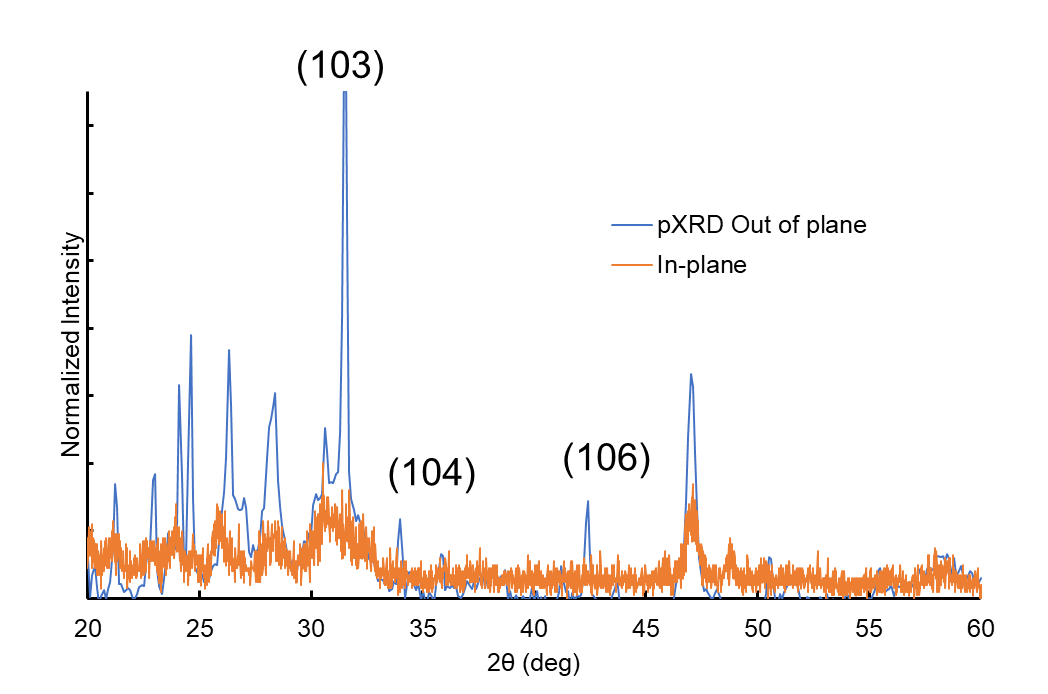


**Figure S2.** Comparison of XRD taken in out of plane (Standard) and in-plane geometry of bilayer films containing Au and CuS nanoparticles in the axial orientation. The indexed peaks have dominant c-components and are less intense in the in-plane measurement compared to the standards out-of-plane measurement, suggesting the axial orientation of the nanoparticles.

a)

b)

525

nm

Au

LSPR

1050 nm

350 nm

1050 nm

CuS LSPR

525

nm

Au

LSPR

1050 nm

350 nm

**Figure S3**. Diagram of Cascaded Sum frequency generation (SFG) and comparison to direct SFG in dual beam mode.

The cascaded SFG process is a two-step mechanism in which two photons of fundamental frequency light are combined in a SHG process whose output then undergoes SFG with another fundamental harmonic photon to produce light at the third harmonic (Figure S3a). This process relies on 3 input photons at frequency ω and thus has a third order with respect to the input beam intensity. To test the feasibility of the process, the two steps in the cascaded process were decoupled, by generating the 2ω independently with a standard nonlinear crystal and recombining that with the split fundamental as demonstrated in Figure 3a. This reduced the process to just the SFG step, as shown in diagram b.

**
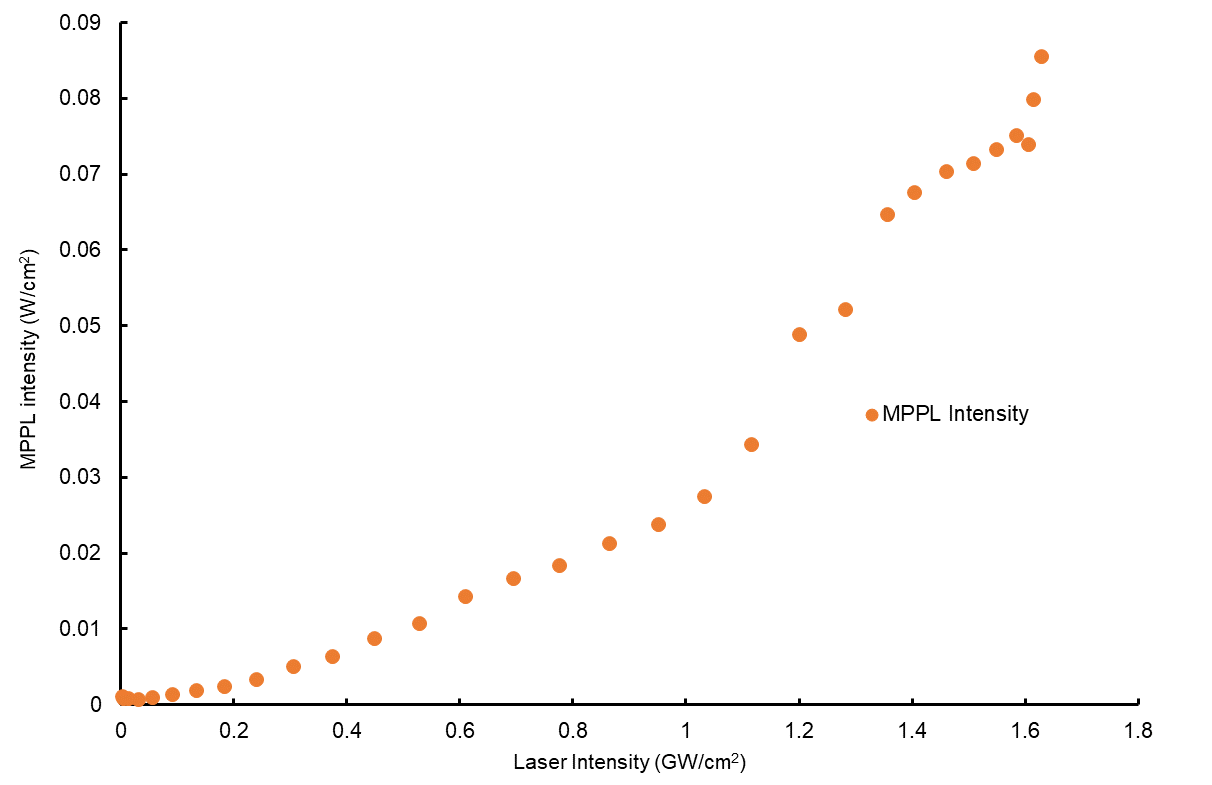
Figure S4.** Measurement of MPPL from dual beam stimulation of Au-CuS films with 50-50 beam splitting. The quantity of MPPL is the broad band intensity of light collected by the PMT (600-300 nm) with the second harmonic (525 nm) filtered out and the THG signal (350 nm) subtracted.


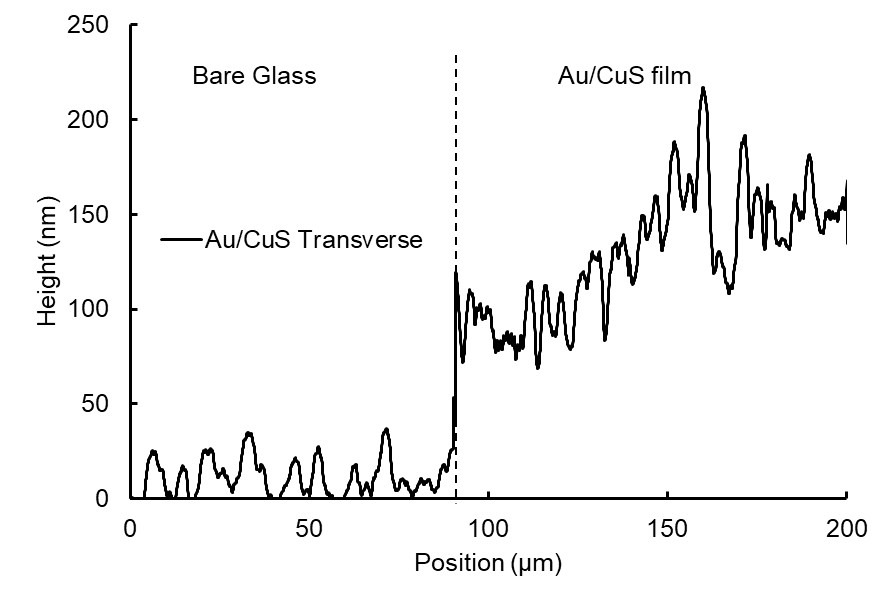


**Figure S5.** Film thickness measurement

Profilometry measurement of film thickness. Low positions (before dotted line) correspond to bare glass where the nanoparticle films have been wiped away. Difference in average height between bare glass and film domains (film height) is 121 nm.

**
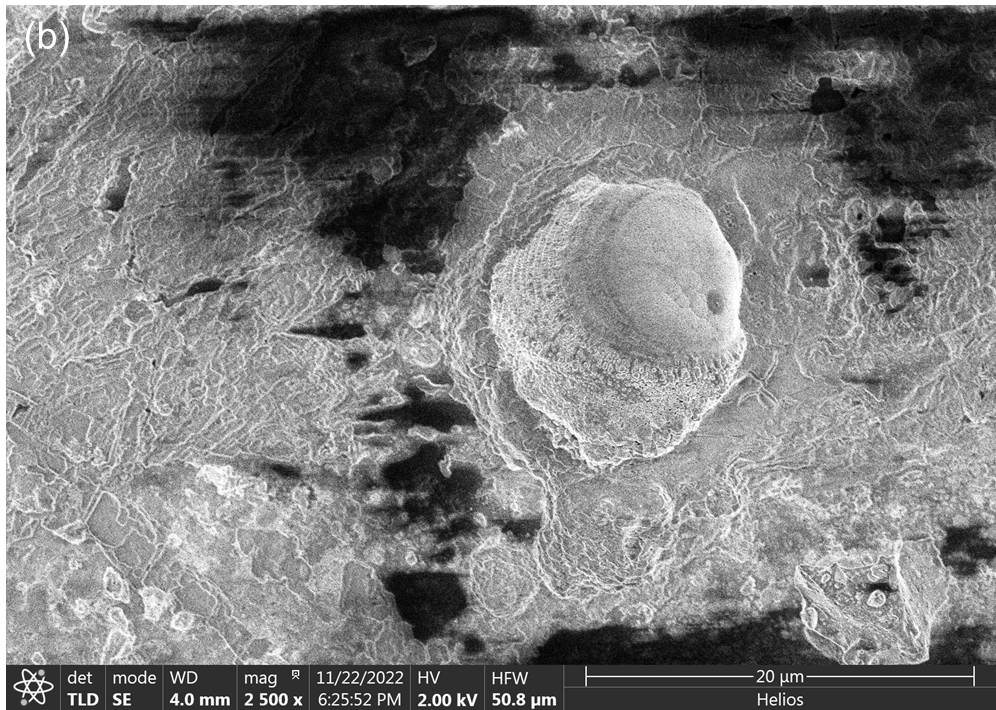

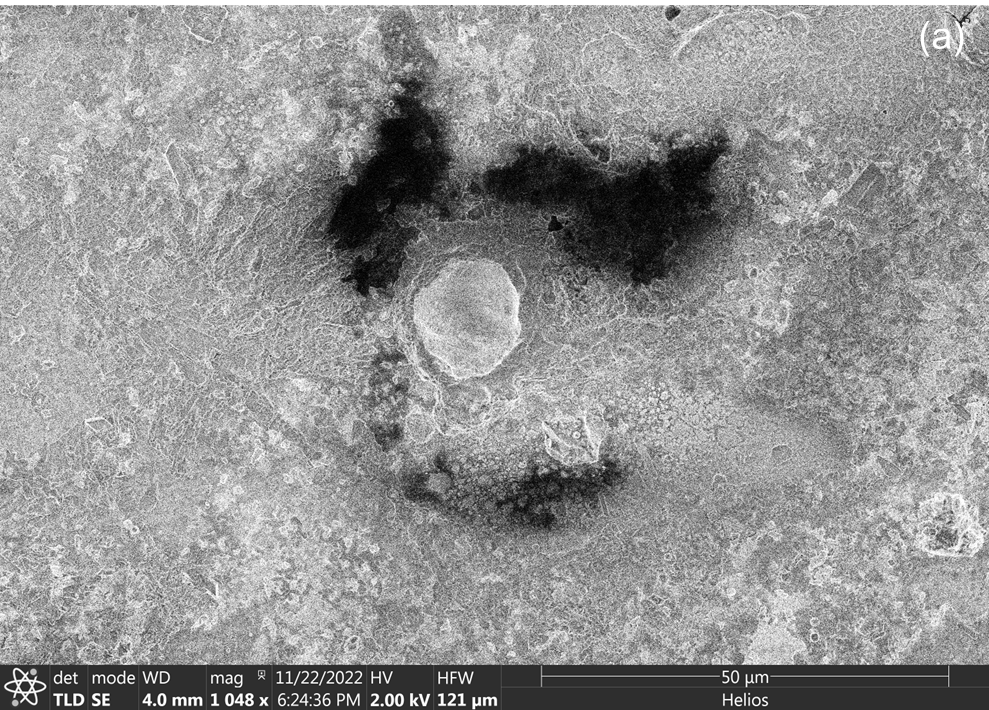
**

**
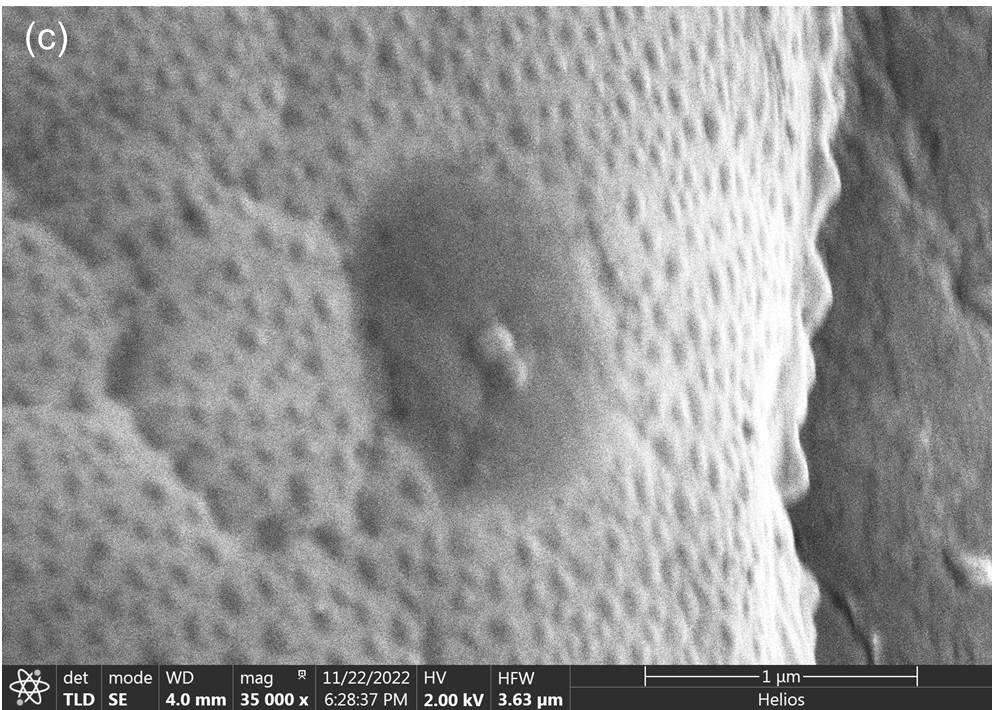

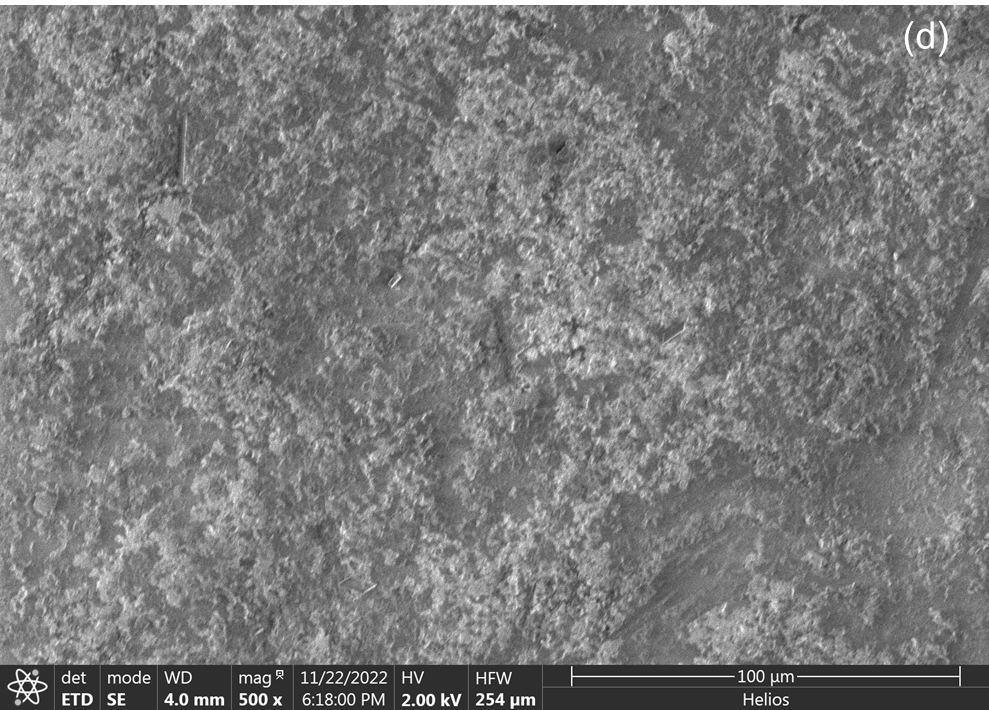

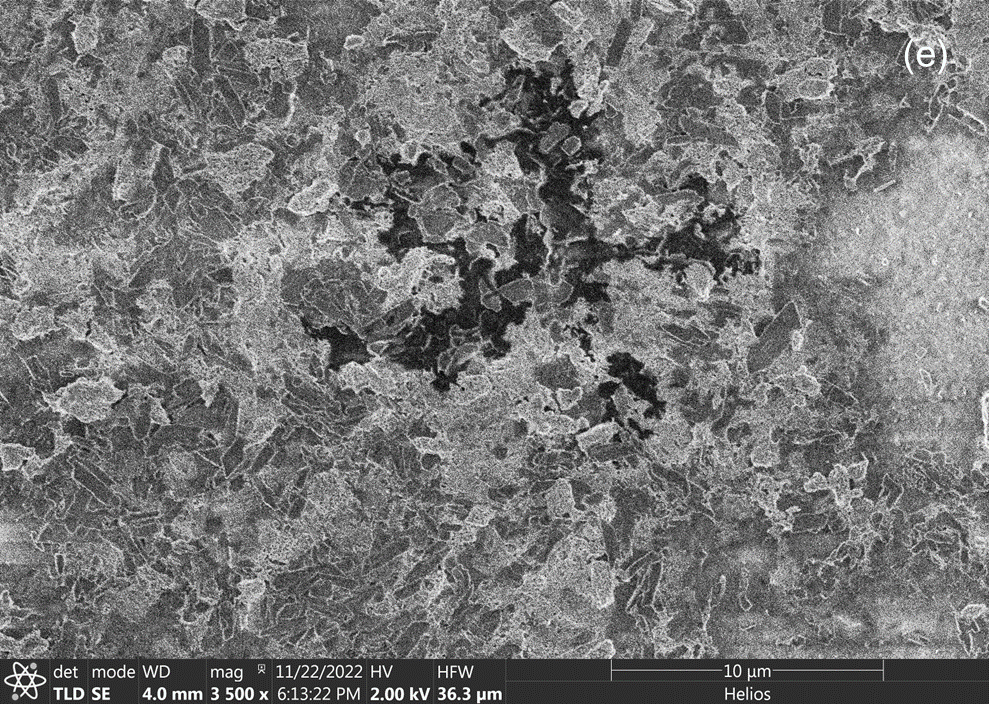

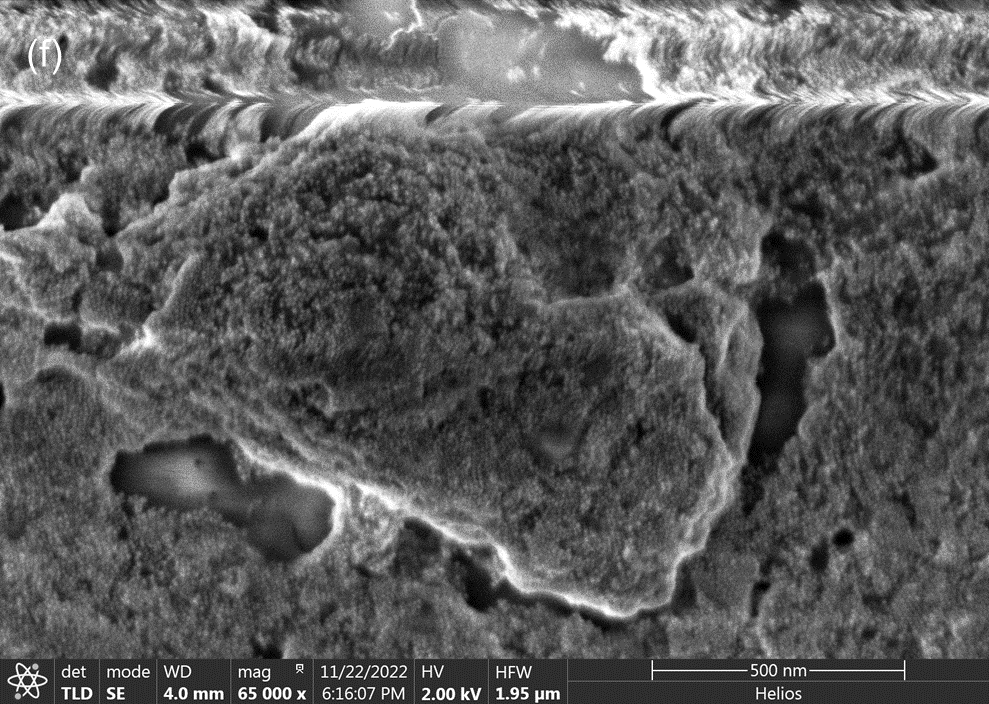
Figure S6.** SEM of Au/CuS bilayer films at (a-c) the site of laser exposure and (e-f) sites without laser exposure

**
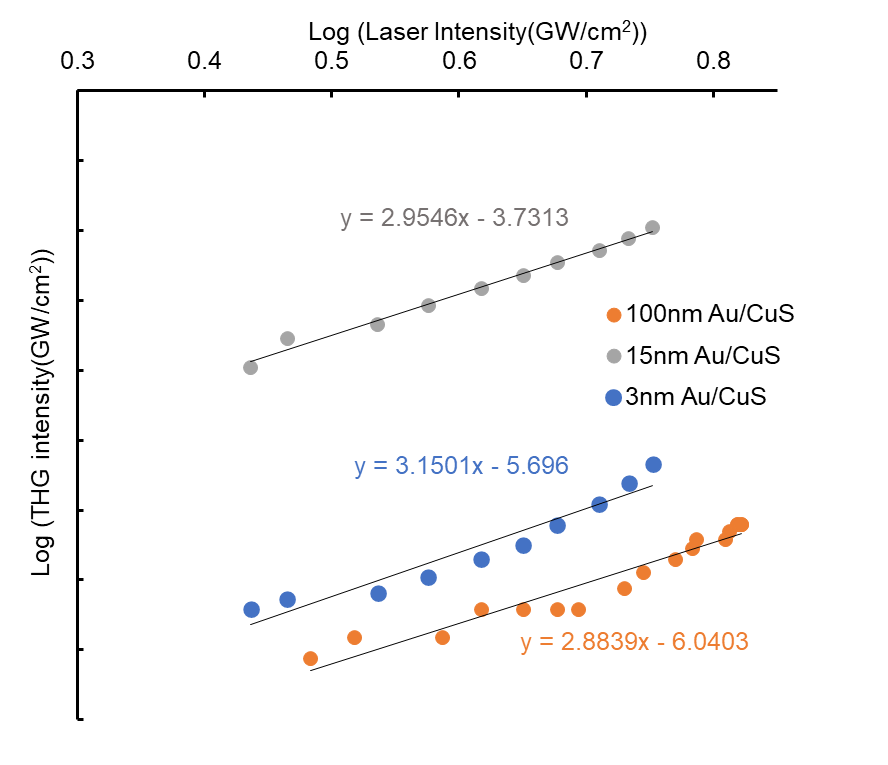

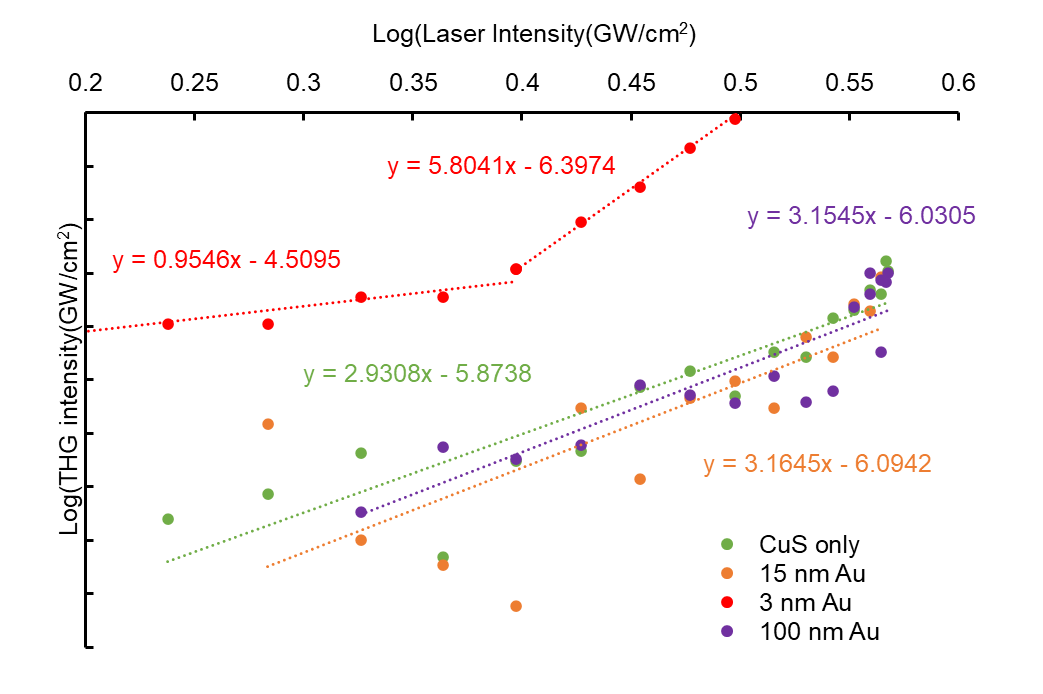
Figure S7.** Log-log plots of data from figures 2 b,c,d of bilayer (Au/CuS) films demonstrating third order dependence. Notably, the 3nm Au nanoparticles feature a sharp transition in response order from 1 to about 6. This is consistent with previous results from nanoparticles in which the harmonic generation and MPPL signals spectrally overlap[1].

**Section S2.** Theory of Third Harmonic Generation in Au and CuS Nanoparticle heterostructure

2.1 SPP Fields in Au and CuS nanoparticles

We consider that the metallic nanohybrid is made of an ensemble of Au nanoparticles (NPs) and CuS NPs. The nanohybrid is deposited on a substrate (i.e., background material) with dielectric constant ϵ_b_. A schematic diagram of the nanohybrid is shown in Figure S8. We know that the free electrons are present on the surface of the Au-NP. These electrons oscillate collectively and produce electron surface plasmons. When a pump field is applied to the Au-NP, photons of the pump field interact with the surface plasma and create quasiparticles called surface plasmon polaritons (SSPs).


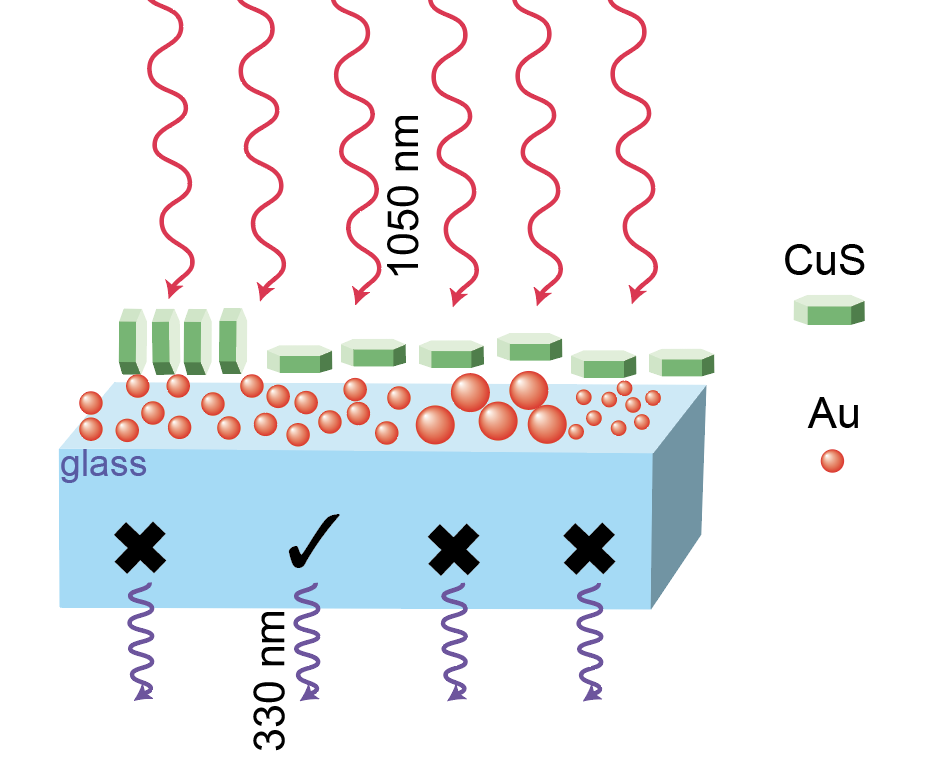


**Figure S8**: Schematic diagram of a hybrid which consists of an ensemble of interacting Au-nanoparticles and CuS nanoparticles.

We know that CuS is a direct band gap semiconductor, and that CuS-NPs are heavily doped p-type semiconductors. The p-type semiconductors have positively charged holes which are free to move in materials. Hence, a CuS-NP has free holes on surface and these holes oscillate collectively and produce hole surface plasmons. When a pump field is applied on the CuS-NP, photons interact with the hole surface plasmons and create surface plasmon polaritons (SSPs).

Let us calculate the SPP electric field produced by the Au-NP and CuS-NP, considering that the Au-NPs are spherical. The dielectric constant of the Au-NP is denoted as $\epsilon_{au}$ . The radius of the Au-NP is taken as $R_{Au}$. We applied a pump field with amplitude $E_{p}$ and frequency $\omega_{p}$in the nanohybrid. The pump field induces a dipole in the Au-NP, and it is denoted as $p_{au}$. This dipole produces the SPP field denoted as $E_{spp}^{Au}$. Solving the Maxwell's equations in the quasi-static approximation [2]–[6] one can find the following expression of $E_{spp}^{au}$ as

$E_{spp}^{Au}=\frac{p_{Au}}{4\pi\in_{0}\in_{b}r^{3}}$ (1)

$P_{au}=\epsilon_{0}\epsilon_{b}V_{Au}g_{l}\zeta_{Au}\left( E_{p} \right) V_{au}=\frac{4}{3}\pi{R_{Au}}^{3}$ (2)

$\zeta_{Au}=\left[ \frac{\in_{Au}-\in_{b}}{\in_{Au}+2\in_{b}} \right]$ (3)

In the eqns. (2), the constant $g_{l}$ is called the polarization parameter and it has values $g_{l}=1$ and $g_{l}=-2$ for $\boldsymbol{p}_{Au}||\boldsymbol{E}_{spp}^{Au}$ and $\boldsymbol{p}_{au}\perp\boldsymbol{E}_{spp}^{Au}$, respectively. Here the $\zeta_{au}$ quantity is called the SPP polarization factor of the Au-NP.

Similarly, one can calculate the SPP field produced by the CuS-NP. We consider that the shape of CuS-NPs is rectangular whose volume is $V_{CuS}=L_{x}L_{y}L_{z}$. The dielectric constant of the CuS-NP is denoted as $\epsilon_{cus}$. The pump field induces a dipole $p_{CuS}$ in the CuS-NP. This dipole produces the following SPP field $E_{spp}^{CuS}$.

$E_{spp}^{CuS}=\frac{p_{CuS}}{4\pi\in_{0}\in_{b}r^{3}}$ (4)

$p_{CuS}=\in_{0}\in_{b}V_{CuS}g_{l}\zeta_{CuS}^{i}\left( E_{p} \right) \zeta_{CuS}^{i}=\frac{\in_{CuS}-\in_{b}}{3\eta_{i}^{CuS}\left( \in_{CuS}-\in_{b} \right)+3\in_{b}} i=x,y,z$ (5)

$\eta_{x}^{CuS}=\frac{1}{2}\int_{0}^{\infty} \frac{ds}{\left( s+L_{x}^{2} \right)^{3/2}\left( s+L_{y}^{2} \right)^{1/2}\left( s+L_{z}^{2} \right)^{1/2}}$ (6)

$\eta_{y}^{CuS}=\frac{1}{2}\int_{0}^{\infty} \frac{ds}{\left( s+L_{x}^{2} \right)^{1/2}\left( s+L_{y}^{2} \right)^{3/2}\left( s+L_{z}^{2} \right)^{1/2}}$ (7)

$\eta_{z}^{CuS}=\frac{1}{2}\int_{0}^{\infty} \frac{ds}{\left( s+L_{x}^{2} \right)^{1/2}\left( s+L_{y}^{2} \right)^{1/2}\left( s+L_{z}^{2} \right)^{3/2}}$ (8)

Where the $\zeta_{CuS}^{i}$ quantity is called the SPP polarization factor of the CuS-NP. Note that it depends on the shape of the sample. For this treatment, the condition in which CuS nanoparticles are oriented with their basal planes parallel to the substrate surface (transverse orientation) is considered. In this orientation, only the x and y shape anisotropy factors are considered, which due to the hexagonal disk shape of the nanoparticle have the same value.

The SPP fields of Au-NS and CuS-NP can be written in the compact forms as follows.

$E_{spp}^{Au}=\Pi_{Au}E_{p}$ $\Pi_{Au}=\frac{V_{Au}g_{l}}{{4\pi r}^{3}}\xi_{Au}$ (9)

$E_{spp}^{Cus}=\Pi_{CuS}E_{p}$ $\Pi_{CuS}=\frac{V_{CuS}g_{l}}{{4\pi r}^{3}}\xi_{CuS}$ (10)

Note that both electric fields depend on r^-3^. The $\Pi_{Au}$ and $\Pi_{CuS}$ parameters are called the SPP coupling constants for Au-NP and CuS-NP, respectively.

Let us calculate the SPP resonance frequency of Au-NP. We consider the following form of the dielectric constant for Au-NP, which is widely used in the plasmonic literature.

$\in_{Au}=\in_{\infty}\left( 1-\frac{\omega_{Au}^{2}}{\omega_{p}(\omega_{p}+i/\tau_{Au})} \right)$ (11)

In the above expression, $\omega_{Au}$ is the plasmon frequency and $\epsilon_{\infty}$ is the dielectric constant of metal when light frequency is very large. Here $\tau_{Au}$ is the decay rate which is responsible for the heat energy loss. Note that the real part of $\epsilon_{Au}$ has a negative value when ${\omega_{p}<\omega}_{Au}$. It is interesting to find that when $\epsilon_{Au}$ has a negative value, the denominator of the polarization factor $\zeta_{Au}$ given in Equation 3 becomes zero at a certain value of the frequency. Let us call this value $\omega_{p}=\omega_{Au}^{res}$ . This means that the polarization and SPP field have a huge value when $\omega_{p}=\omega_{Au}^{res}$.

**2.2 THG in Au and CuS nanohybrid**

We have established that the Au-NP has one SPP resonance $\omega_{Au}^{res}$. Therefore, we treat the Au-NP as a two-level system whose ground state is denoted as |1>, and its virtual excited state as |2>. The frequency difference between levels |1> and |2> is expressed as $\omega_{Au}^{res}$. A schematic diagram of two-level system is shown in Figure S9.


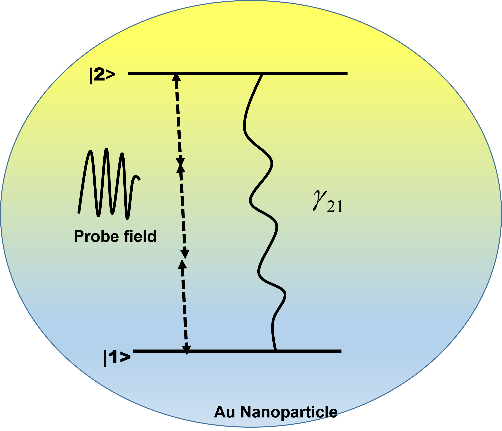


**Figure S9**: A schematic diagram for a two-level Au-NP is plotted. Energy levels are denoted as |1> and |2>. To study the THG, the pump field is applied to the $1\rangle\leftrightarrow|2\rangle$ transition.

.

To study the nonlinear properties in the Au-NP, we calculate the third order susceptibility, $\chi_{Au}^{(3)}$ of the Au nanoparticles. We know that the third order susceptibility is responsible for the THG. Following the method of references,[7],[8] the expression of the third order polarization, $P_{Au}^{(3)}$ , is given as

$P_{Au}^{(3)}=\in_{0}\chi_{Au}^{(3)}E_{p}E_{p}E_{p}$ (12)

Where $\chi_{Au}^{(3)}$ is the third order expressions of the susceptibility $\chi_{Au}$. Following the method of references,[9] the polarization of the Au nanoparticles can also be expressed in terms of the quantum density matrix operator (ρ) as follows.

$P_{Au}^{(3)}=2\frac{p_{Au}}{V_{Au}}\left[ \rho_{Au}^{(3)}+h.c. \right]$ (13)

Here $p_{Au}$ is the matrix element of the dipole moment of the Au nanoparticle and $\varrho_{Au}^{(3)}=\varrho_{21}^{(3)}$ is the third matrix element of density matrix operator $\rho_{au}$ between transition $|1\rangle\leftrightarrow|2\rangle$, and *h.c.* stands for the Hermitian conjugate.

Comparing Equations 12 and 13 and we found the relation between the susceptibility and the density matrix elements as follows.

$\chi_{Au}^{(3)}=\frac{2p_{Au}\left( \rho_{Au}^{(3)} \right)+h.c.}{\in_{0}V_{Au}E_{p}E_{p}E_{p}}$ (14)

We found that the third order susceptibility depends on the third order density matrix elements.

**2.3 Enhancement of THG in Au-NP due to CuS-NP**

We consider that the Au and CuS nanoparticles are present together to study the effect of CuS nanoparticles on the THG intensity of Au nanoparticles. Now, we calculate the third order density matrix element, $\rho_{Au}^{(3)}$ which appears in the expression of the susceptibility.

To calculate the density matrix element, we need to know what electric fields are incident on the Au nanoparticles. The pump field with amplitude $E_{p}$ acts on the Au nanoparticles, while the CuS emits an electric field of the surface plasmon polariton, $E_{spp}^{CuS}$. Therefore, provided that the nanoparticles are spatially close, the SPP field also acts on the Au nanoparticles. Hence, there are two electric fields incident on the Au nanoparticle i.e., $E_{p}$ and $E_{spp}^{CuS}$. The interaction of SPP field with the dipole of the Au nanoparticle is a dipole-dipole interaction.

Using the dipole and rotating wave approximations the interaction Hamiltonian is

$H_{Au}=H_{Au-probe}+H_{DDI}^{Au}$ (15) $H_{Au-probe}=\hbar\Lambda_{Au}\Omega_{p}\sigma_{21}+h.c.H_{DDI}^{Au}=\hbar\Lambda_{Au}\Omega_{CuS}\sigma_{21}+h.c.$ (16)

$\Omega_{P}=\frac{\mu_{21}E_{p}}{\hbar} \Omega_{CuS}=\frac{\mu_{21}E_{spp}^{CuS}}{\hbar}=\Pi_{CuS}\Omega_{p} \Lambda_{Au}=\frac{4\pi\in_{0}\in_{b}R_{Au}^{3}g_{l}\zeta_{Au}\hbar\Omega_{P}}{\mu_{21}^{2}}$ (17)

where h.c. stands for the Hermitian conjugate. Here $\sigma_{21}=|2><|1|$ is the SPP polariton creation operator. The parameter $\Omega_{p}$ and $\Omega_{CuS}$ are the Rabi frequencies associated with the pump and the SPP fields, respectively. The first term in the Hamiltonian is the polariton and photon interaction. The second term is the DDI between Au-NP and CuS-NP.

Density Matrix Method: Following established methods and with the Hamiltonian of Equation (15), the equations of the motion for density matrix elements are

$$\frac{d\rho_{11}}{dt}=+\gamma_{21}\rho_{22}+i\Lambda_{Au}\Omega_{p}\left( \rho_{12}-\rho_{21} \right)+i\Lambda_{Au}\Omega_{CuS}\left( \rho_{12}-\rho_{21} \right)$$

$$\frac{d\rho_{22}}{dt}=-\left( \gamma_{21} \right)\rho_{22}-i\Lambda_{Au}\Omega_{p}\left( \rho_{12}-\rho_{21} \right)-i\Lambda_{Au}\Omega_{CuS}\left( \rho_{12}-\rho_{21} \right)$$

$\frac{d\rho_{21}}{dt}=-d_{21}\rho_{21}+i\Lambda_{Au}\Omega_{p}(\rho_{22}-\rho_{11})+i\Lambda_{Au}\Omega_{CuS}(\rho_{22}-\rho_{11})$ (18)

where

$d_{21}=\delta_{21}+i\gamma_{21} \delta_{21}=\omega_{Au}^{res}-3\omega_{p}$ (19)

Here $\delta_{21}$ is called the field detuning. The physical quantity $\gamma_{21}$ is the transition rate for transition $|2\rangle\leftrightarrow|1\rangle$. Note that we use notation $\rho_{21}^{(3)}=\rho_{Au}^{(3)}$.

The THG density matrix element $\rho_{Au}^{(3)}=\rho_{21}^{(3)}$ is calculated in the third order in $\Omega_{P}^{3}, {\Omega_{P}^{2}\Omega}_{CuS}$, ${\Omega_{CuS}^{2}\Omega}_{P}$, and $\Omega_{CuS}^{3}$, by solving Equation (18) in the steady sate. After some mathematical manipulations we obtained the following analytical expression for $\rho_{Au}^{(3)}$:

$\rho_{Au}^{(3)}=\rho_{ppp}^{Au}+\rho_{pps}^{Au}+\rho_{pss}^{Au}+\rho_{sss}^{Au}$ (20)

The density matrix elements appearing in Equation (20) are

$$\rho_{ppp}^{Au}=\left( \Lambda_{Au}\Omega_{p} \right)^{3}F_{ppp}^{Au}=\Lambda_{Au}^{3}\Omega_{p}^{3}F_{ppp}^{Au}$$

$$\rho_{pps}^{Au}=\left( \Lambda_{Au}\Omega_{p} \right)^{2}\left( \Lambda_{Au}\Omega_{CuS} \right).3F_{ppp}^{Au}=\Lambda_{Au}^{3}\Omega_{p}^{2}\Omega_{CuS}.3F_{ppp}^{Au}$$

$$\rho_{pss}^{Au}=\left( \Lambda_{Au}\Omega_{p} \right)\left( \Lambda_{Au}\Omega_{CuS} \right)^{2}.3F_{ppp}^{Au}=\Lambda_{Au}^{3}\Omega_{p}\Omega_{CuS}^{2}.3F_{ppp}^{Au}$$

$$\rho_{sss}^{au}=\left( \Lambda_{Au}\Omega_{CuS} \right)^{3}F_{ppp}^{Au}=\Lambda_{Au}^{3}\Omega_{CuS}^{3}.F_{ppp}^{Au}$$

$\rho_{ppp}^{Au}=\Omega_{p}^{3}\Lambda_{Au}^{3}F_{ppp}^{Au} \rho_{pps}^{Au}=\Lambda_{Au}^{3}\Omega_{p}^{2}\Omega_{CuS}.3F_{ppp}^{Au}$ (21)

$\rho_{pss}^{Au}=\Lambda_{Au}^{3}\Omega_{p}\Omega_{CuS}^{2}.3F_{ppp}^{Au}\rho_{sss}^{Au}=\Lambda_{Au}^{3}\Omega_{CuS}^{3}F_{ppp}^{Au} F_{ppp}^{Au}=\frac{4iRe \left( d_{21} \right)}{\left( \gamma_{21} \right)d_{21}d_{21}(d_{21})^{*}}$ (22)

We evaluate the third order susceptibility $\chi_{Au}^{(3)}$ by substituting the expression for $\varrho_{Au}^{(3)}$ from Equation (20) into Equation (14) to obtain

$\chi_{Au}^{(3)}=\chi_{ppp}^{Au}+\chi_{pps}^{Au}+\chi_{pss}^{Au}+\chi_{sss}^{Au}$ (23)

where:

$\chi_{ppp}^{Au}=\frac{2\mu_{21}^{3}p_{Au}}{V_{Au}\in_{0}\hbar^{3}}\left( \Lambda_{Au}^{3}F_{ppp}^{Au} \right) \chi_{pps}^{Au}=\frac{6\mu_{21}^{3}p_{Au}}{V_{Au}\in_{0}\hbar^{3}}\left( \Lambda_{Au}^{3}\Pi_{CuS}F_{ppp}^{Au} \right)$ (24)

$\chi_{pss}^{Au}=\frac{6\mu_{21}^{3}p_{Au}}{V_{Au}\in_{0}\hbar^{3}}\left( \Lambda_{Au}^{3}\Pi_{CuS}^{2}F_{ppp}^{Au} \right) \chi_{pps}^{Au}=\frac{2\mu_{21}^{3}p_{Au}}{V_{Au}\in_{0}\hbar^{3}}\left( \Lambda_{Au}^{3}\Pi_{CuS}^{3}F_{ppp}^{Au} \right)$ (25)

**The THG intensity**: We calculate the THG intensity for the Au-NP as follows.

$I_{THG}^{Au}=\frac{1}{2}\sqrt{\frac{\in_{0}}{\mu_{0}}}n_{3}\left| E_{THG}^{Au} \right|^{2}=\frac{\in_{0}c}{2}n_{3}\left| E_{THG}^{Au} \right|^{2}$ (26)

Here $E_{THG}^{Au}$ is the THG electric field emitted by the Au nanoparticle.

The intensity of THG can be calculated by solving the nonlinear Maxwell equations. One can find the following expression of the $E_{THG}^{Au}$ field.

$E_{THG}^{Au}=i\frac{6\omega_{3}L}{c}\left\{ \begin{aligned} &\chi_{ppp}^{Au}E_{p}E_{p}E_{p}+\chi_{pps}^{Au}E_{p}E_{p}E_{CuS} \\ &+\chi_{ps}^{Au}E_{p}E_{CuS}E_{CuS}+\chi_{sss}^{Au}E_{CuS}E_{CuS}E_{CuS} \end{aligned} \right\}$ (27)

Inserting Equation 27 into Equation 26 we get the expression for the THG intensity.

$I_{THG}^{Au}=\frac{18\in_{0}\omega_{3}^{2}L^{2}n_{3}}{c}\left\{ \begin{aligned} &\left| \chi_{ppp}^{Au} \right|^{2}\left| E_{p}^{3} \right|^{2}+\left| \chi_{pps}^{Au} \right|^{2}\left| E_{p}^{2}E_{CuS} \right|^{2} \\ &+\left| \chi_{pss}^{Au} \right|^{2}\left| E_{p}E_{CuS}^{2} \right|^{2}+\left| \chi_{pss}^{Au} \right|^{2}\left| E_{CuS}^{3} \right|^{2} \end{aligned} \right\}$ (28)

Note that the cross terms are neglected because they correspond to coherences. These were neglected due to the short dephasing time of the plasmon resonance with respect to the exciting pulse, with a dephasing time on the order of 10 fs, and pulse width of 177fs. This means that the plasmon resonances lose phase coherence due to electron-electron collisions and thermalize very quickly after the end of the pulse. Thus, coherence effects have been neglected, congruous with previous treatments of similar systems [5],[10],[11].

It is possible to include the effect of coherence in our formulation in our future works whether this effect is important or not important for the present paper. However, the inclusion of coherence will make the expression of the THG intensity very complicated and challenging. An analytical expression for the THG intensity could not be achieved. One of the aims of the present theory was to derive a simple expression of the THG intensity so that experimentalists working in the plasmonic field can compare their experiments with the current theory.

We express the $E_{p}$ and $E_{CuS}$ in terms of pump intensity $I_{p}$ and SPP intensity $I_{CuS}$ as follows

$I_{p}=\frac{\in_{0}c}{2}n_{3}\left| E_{p} \right|^{2} I_{CuS}=\frac{\in_{0}c}{2}n_{3}\left| E_{spp}^{CuS} \right|^{2}$ (29)

Now, we insert Equations 24, 25, and 29 into Equation 28 to get an expression of the THG intensity.

$I_{THG}^{Au}=\alpha_{cst}^{Au}\Lambda_{Au}^{6}\left| F_{ppp}^{Au} \right|^{2}\left\{ {I_{p}}^{3}+9{\Pi_{CuS}}^{2}{I_{p}}^{2}I_{CuS}+9{\Pi_{CuS}}^{4}{I_{CuS}}^{2}I_{p}+{\Pi_{CuS}}^{6}{I_{CuS}}^{3} \right\}$ (30)

Where:

$\alpha_{THG}^{Au}=\frac{144\omega_{3}^{2}L^{2}\mu_{21}^{6}p_{Au}^{2}}{\in_{0}^{4}V_{Au}^{2}c^{4}\hbar^{6}n_{3}^{2}}$ (31)

One can see from Equation 30 that there is an enhancement in THG due to the presence of the CuS nanoparticles.

**2.4 Enhancement in THG Intensity in CuS-NP due to Au-NPs**

Next, we study the effect of Au nanoparticles on the THG intensity emitted by CuS-NP. We find that Au-NP emits the surface-plasmon polariton electric field, $E_{spp}^{Au}$. The CuS-NP is interacting with Au-NP via $E_{spp}^{Au}$ field. This interaction is nothing but the dipole-dipole interaction. The interaction Hamiltonian for CuS nanoparticle in the dipole and rotating wave approximation can be written as follows.

$H_{Cus}=H_{Cus-probe}+H_{DDI}^{Cus}$

$$H_{Cus-probe}=\hbar\Lambda_{CuS}\Omega_{p}\sigma_{21}+h.c.$$

$H_{DDI}^{CuS}=\hbar\Lambda_{Cus}\Omega_{Au}\sigma_{21}+h.c$ (32)

Where the physical parameters appearing in Equation 32 are found as

$\Omega_{Au}=\frac{\mu_{21}E_{spp}^{Au}}{\hbar}=\Pi_{Au}\Omega_{P} \Lambda_{CuS}=\frac{4\pi\in_{0}\in_{b}V_{CuS}g_{l}\zeta_{CuS}\hbar\Omega_{P}}{\mu_{21}^{2}}$ (33)

Note that $H_{DDI}^{CuS}=H_{DDI}^{Au}$. The first term in the interaction Hamiltonian between the SPP polariton with the pump photon. The second term the dipole-dipole interaction between CuS-NP and Au-NP.

Following the density matrix method of Au-NP, we found the third order density matrix $\varrho_{21}^{(3)}=\varrho_{CuS}^{(3)}$ for CuS-NP as

$\rho_{CuS}^{(3)}=\rho_{ppp}^{CuS}+\rho_{pps}^{CuS}+\rho_{pss}^{CuS}+\rho_{sss}^{CuS}$ (34)

The density matrix elements appearing in Equation 34 are found as

$\rho_{ppp}^{CuS}=\Omega_{p}^{3}\Lambda_{CuS}^{3}F_{ppp}^{CuS} \rho_{pps}^{CuS}={3\Omega}_{p}^{3}\Lambda_{CuS}^{3}\Pi_{Au}F_{ppp}^{CuS}$ (35) $\rho_{pss}^{CuS}=3\Omega_{p}^{3}\Lambda_{CuS}^{3}\Pi_{Au}^{2}F_{ppp}^{CuS} \rho_{sss}^{CuS}=\Omega_{p}^{3}\Lambda_{CuS}^{3}\Pi_{Au}^{3}F_{ppp}^{CuS}$ (36)

Finally, with the help of Equation 35, 36, the THG intensity emitted by the CuS-NP is calculated as follows.

$I_{THG}^{Cus}=\alpha_{cst}^{Cus}\Lambda_{CuS}^{6}\left| F_{ppp}^{Cus} \right|^{2}\left\{ {I_{p}}^{3}+9{\Pi_{Au}}^{2}{I_{p}}^{2}I_{Au}+9{\Pi_{Au}}^{4}{I_{Au}}^{2}I_{p}+{\Pi_{Au}}^{6}{I_{Au}}^{3} \right\}$ (37)

where

$\alpha_{THG}^{CuS}=\frac{144\omega_{3}^{2}L^{2}\mu_{21}^{6}p_{CuS}^{2}}{\in_{0}^{4}V_{CuS}^{2}c^{4}\hbar^{6}n_{3}^{2}}$ (38)

We found that the THG intensity of CuS-NP is enhanced due to the presence of Au nanoparticles.

**2.5 THG Intensity in Au and CuS nanohybrid**

The THG intensity emitted by the Au-NP and CuS-NP hybrid can be obtained by adding the intensities of the Au and CuS nanoparticles. The THG intensity for the hybrid system is found as

$I_{THG}^{hybrid}=I_{THG}^{Au}+I_{THG}^{CuS}$ (39)

Substituting the expression for $I_{THG}^{Au}$ and $I_{THG}^{CuS}$ from Equations 30 and 37 into Equation 39, we get

$I_{THG}^{hybrid}=\left[ \alpha_{cst}^{Au}\Lambda_{Au}^{6}\left| F_{ppp}^{Au} \right|^{2}\left\{ {I_{p}}^{3}+9{\Pi_{CuS}}^{2}{I_{p}}^{2}I_{CuS}+9{\Pi_{CuS}}^{4}{I_{CuS}}^{2}I_{p}+{\Pi_{CuS}}^{6}{I_{CuS}}^{3} \right\}+\alpha_{cst}^{Cus}\Lambda_{CuS}^{6}\left| F_{ppp}^{CuS} \right|^{2}\left\{ {I_{p}}^{3}+9{\Pi_{Au}}^{2}{I_{p}}^{2}I_{Au}+9{\Pi_{Au}}^{4}{I_{Au}}^{2}I_{p}+{\Pi_{Au}}^{6}{I_{Au}}^{3} \right\} \right]$ (40)

We predicted that the hybrid intensity depends on the dipoles and SPP coupling constant of the Au and CuS nanoparticles.

The expression of the THG intensity can be further written in the compact form as follows

$I_{THG}=C_{au}\left\{ {I_{p}}^{3}+9{\Pi_{CuS}}^{2}{I_{p}}^{2}I_{CuS}+9{\Pi_{CuS}}^{4}{I_{CuS}}^{2}I_{p}+{\Pi_{Cus}}^{6}{I_{CuS}}^{3} \right\}+C_{CuS}\left\{ {I_{p}}^{3}+9{\Pi_{Au}}^{2}{I_{p}}^{2}I_{Au}+9{\Pi_{Au}}^{4}{I_{Au}}^{2}I_{p}+{\Pi_{Au}}^{6}{I_{Au}}^{3} \right\}$ (41)

Where

$C_{CuS}=\alpha_{cst}^{CuS}\Lambda_{CuS}^{6}\left| F_{ppp}^{CuS} \right|^{2} C_{Au}=\alpha_{cst}^{Au}\Lambda_{Au}^{6}\left| F_{ppp}^{Au} \right|^{2}$ (42)

Note that unit of $C_{Au}$ and $C_{CuS}$ is (W/m^2^)^-2^. The intensity parameters $I_{p}$, $I_{Au}$and $I_{CuS}$ have unit (W/m^2^). On the other hand, the SPP coupling parameters $\Pi_{Au}$ and $\Pi_{CuS}$ are unitless.

**2.6 SHG intensity of Au-NP alone and CuS-NP alone**

We can calculate the THG intensity emitted from Au nanoparticles alone from Equation 40 by putting $\Pi_{CuS}=0$ and $\Lambda_{CuS}=0$ to obtain

$I_{THG}^{Au}=\alpha_{cst}^{Au}\Lambda_{Au}^{3}\left| F_{ppp}^{Au} \right|^{2}.I_{p}^{3}$ (43)

Similarly, the THG intensity emitted from CuS nanoparticle alone can be calculated from Equation 40 by putting $\Pi_{Au}=0$ and $\Lambda_{Au}=0$.

$I_{THG}^{CuS}=\alpha_{cst}^{CuS}\Lambda_{CuS}^{3}\left| F_{ppp}^{CuS} \right|^{2}I_{p}^{3}$ (44)

**2.7 Comparison of Theory and Experiments**

**
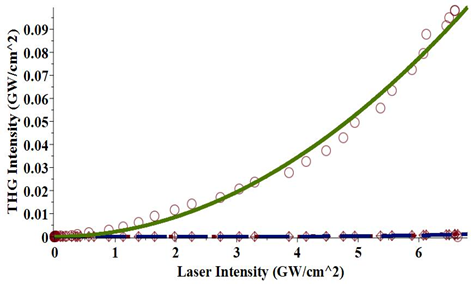
**

**Figure S10**: A comparison between the theory and experiments for the 15 nm sample. The experimental points are shown in circles for the Au-CuS sample, crosses for the Au sample and diamonds for the CuS sample.  The dashed line and dotted lines are for the Au-only and the CuS-only samples, respectively. The dash-dotted line adds the THG intensities from single layers of Au and CuS nanoparticles.

The solid line in Figure S10 is the fit for the Au-CuS sample, derived from Equation 40. The SPP coupling parameter values that result from the fitting procedure are Π_CuS_ = 3.1 and Π_Au_ = 0, corresponding to single-beam excitation at 1050 nm as in the experiment. The theoretical value Π_CuS_ = 3.1 is used to calculate the output intensity of the third harmonic as shown in Figure S11.

**
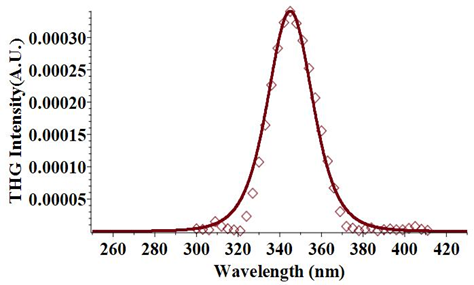
**

**Figure S11**: Output intensity of the THG signal with 1050 nm fundamental input. Experimentally measured points are shown in diamonds. The solid line is theoretical calculation from input beam properties.

By substituting the following physical constants into Equations 5, 6 and 10,

ε_CuS_ = 5.76, ε_b_ = 1.69, L_x_ = L_y_ = 15 nm, $\frac{V_{CuS}}{4\pi r^{3}}= 2.1$

where the ratio $\frac{V_{CuS}}{4\pi r^{3}}= 2.1$ is based on the estimated packing fraction of the nanoparticles on the surface in Figure 1d, we calculate the value of Π_CuS_ to be 1.7; within the limits of accuracy with which we can estimate the physical parameters, this is reasonably close to the value derived from fitting the experimental data.

**
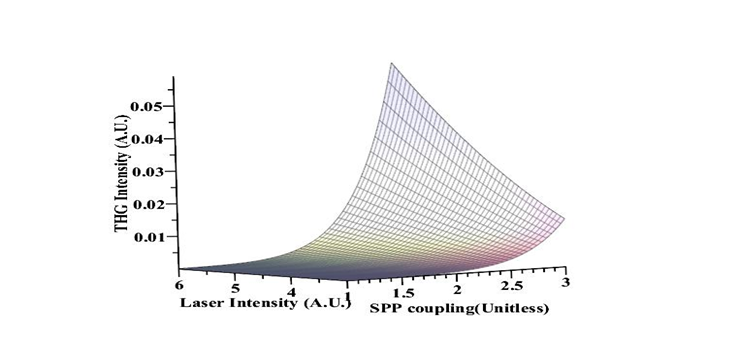
**

**Figure S12**: A plot of the THG intensity as a function of the input intensity and the SPP coupling.

**
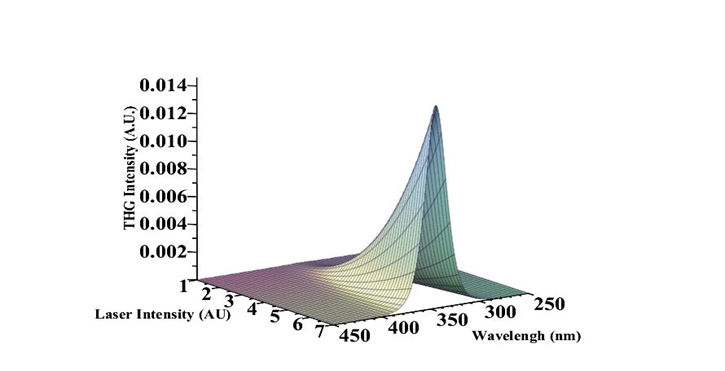
**

**Figure S13**: A plot of the THG intensity as a function of the input intensity and wavelength.

Figures S12 and S13 show that the dramatic increase in THG as a function of pump intensity and SPP coupling (S12) and wavelength (S13) calculated from theory are consistent with the experimental observations. In particular, the pump-induced broadening of the third harmonic signal is what one would expect for the range of experimentally sampled pump intensities. However, the strong dependence of THG intensity at the highest intensities (S12) also hints at the enormous influence of the SPP coupling constant – an issue that should be explored as a function of bilayer gap length.

**Supplemental References**

[1] N. J. Spear, K. A. Hallman, E. A. Hernández-Pagán, R. B. Davidson, et al. "Enhanced Broadband and Harmonic Upconversion from Coupled Semiconductor and Metal Nanoparticle Films," *ACS Appl. Nano Mater.*, vol. 3, no. 4, pp. 3144–3150, 2020. https://doi.org/10.1021/acsanm.0c00064.

[2] B. Novotny, Lukas, Hecht. "Principles of Nano-Optics"; Cambridge University Press: Cambridge. https://doi.org/https://doi.org/10.1017/CBO9780511813535.

[3] W. Sarid, Dror; A. Challener. "Modern Introduction to Surface Plasmons"; Cambridge University Press: Cambridge, UK. https://doi.org/https://doi.org/10.1017/CBO9781139194846.

[4] M. Singh. "Electronic, Photonic, Polaritonic, and Plasmonic Materials"; Wiley Custom: Toronto.

[5] M. R. Singh, S. Yastrebov. "Switching and Sensing Using Kerr Nonlinearity in Quantum Dots Doped in Metallic Nanoshells," *J. Phys. Chem. C*, vol. 124, no. 22, pp. 12065–12074, 2020. https://doi.org/10.1021/acs.jpcc.0c02122.

[6] M. R. Singh. "Theory of All-Optical Switching Based on the Kerr Nonlinearity in Metallic Nanohybrids," *Phys. Rev. A*, vol. 102, no. 1, pp. 1–10, 2020. https://doi.org/10.1103/PhysRevA.102.013708.

[7] A. Hanamura, Eiichi; Kawabe, Yutaka; Yamanaka. "Quantum Nonlinear Optics"; Springer US.

[8] R. Boyd. "Nonlinear Optics", Third Edit.; Academic Press: Burlington, MA.

[9] M. O. Scully, M. S. Zubairy. "Quantum Optics"; Cambridge University Press: Cambridge, UK. https://doi.org/10.1017/CBO9780511813993.

[10] M. R. Singh, G. Brassem, S. Yastrebov. "Optical Quantum Yield in Plasmonic Nanowaveguide," *Nanotechnology*, vol. 32, no. 13, 2021. https://doi.org/10.1088/1361-6528/abd05d.

[11] X. Han, K. Wang, P. D. Persaud, X. Xing, et al. "Harmonic Resonance Enhanced Second-Harmonic Generation in the Monolayer WS2-Ag Nanocavity," *ACS Photonics*, vol. 7, no. 3, pp. 562–568, 2020. https://doi.org/10.1021/acsphotonics.9b01499.
